# Marine Fungi: A Source of Potential Anticancer Compounds

**DOI:** 10.3389/fmicb.2017.02536

**Published:** 2018-01-05

**Authors:** Sunil K. Deshmukh, Ved Prakash, Nihar Ranjan

**Affiliations:** ^1^TERI–Deakin Nano Biotechnology Centre, The Energy and Resources Institute, New Delhi, India; ^2^Department of Biotechnology, Motilal Nehru National Institute of Technology, Allahabad, India

**Keywords:** marine fungi, deep sea fungi, cytotoxic compounds, mangroove associated fungi, sponge associated fungi

## Abstract

Metabolites from marine fungi have hogged the limelight in drug discovery because of their promise as therapeutic agents. A number of metabolites related to marine fungi have been discovered from various sources which are known to possess a range of activities as antibacterial, antiviral and anticancer agents. Although, over a thousand marine fungi based metabolites have already been reported, none of them have reached the market yet which could partly be related to non-comprehensive screening approaches and lack of sustained lead optimization. The origin of these marine fungal metabolites is varied as their habitats have been reported from various sources such as sponge, algae, mangrove derived fungi, and fungi from bottom sediments. The importance of these natural compounds is based on their cytotoxicity and related activities that emanate from the diversity in their chemical structures and functional groups present on them. This review covers the majority of anticancer compounds isolated from marine fungi during 2012–2016 against specific cancer cell lines.

## Introduction

Marine fungi are important source of secondary metabolites useful for the drug discovery purposes. Even though marine fungi are less explored in comparison to their terrestrial counterparts, a number of useful hits have been obtained from the drug discovery perspective adding to their importance in the natural product discovery (Molinski et al., 2009; Butler et al., 2014), which have yielded a wide range of chemically diverse agents with antibacterial, antiviral and anticancer properties in animal systems. Starting with the celebrated example of cephalosporins, marine fungi have provided unique chemical skeletons that could be used to develop drugs of clinical importance (Bhadury et al., 2006; Saleem et al., 2007; Javed et al., 2011; Sithranga and Kathiresan, 2011). Fungi, in general, have been generous source of drugs as evidenced by the isolation of many drugs in use such as paclitaxel, camptothecin, vincristine, torreyanic acid and cytarabine to name a few. In this light, marine are important not just from the perspective of new drugs but also as a source of new scaffolds that can be modified further to obtain the desired action. Despite significant progress in the drug discovery that has provided treatment for some major ailments, minor infections, and epidemics; new drugs are required to combat global resistance to drugs for existing diseases and new infections that have been reported in recent times (such as SARS, dengue and Zika viruses). In addition to drug resistance in diseases such as tuberculosis and malaria, cancer & HIV-AIDS (Passaes and Sáez-Cirión, 2014) have been biological targets with limited success toward therapeutics development.

In addition to terrestrial sources, oceans have been a huge reservoir of a variety of biologically active compounds, which have often been the resulting metabolite of marine life (König et al., 2006; Chen G. et al., 2014; Agrawal et al., 2016; Deshmukh et al., 2017). Though, why marine fungi produce such complex and diverse set of metabolites in not fairly understood, it is largely assumed that they play key roles in chemical defense and communication. The biosynthesis of these metabolites in dependent on ecological, physical and biological factors and, therefore, small changes in these conditions can generate entirely new set of metabolites (Pejin and Maja, 2017). The contribution of marine based therapeutics can be gauged from the fact that during 1981–2002, more than half of the FDA approved drugs had originated from marine life. Most of the marine based drugs have come from invertebrates (sponges, tunicates, mollusks, and bryozoans); two-thirds of which, belong to the class of non-ribosomal peptides. Some of these are already in the market (Polymixin B, pristinamycin, gramicidin, vancomycin, bleomycin, actinomycin D) as antibiotic and anti-cancer agents while several others are in clinical trials (Manoalide, discodermolide) (Singh et al., 2008). In this regard, there have not been many reports of drugs from marine fungi that are used clinically which can be partly attributed to lack of systematic and comprehensive approaches as well as lack of optimization which has precluded a large number of potential hits from becoming actual drugs. Therefore, metabolites marine fungi constitute a group of under-represented resource for discovering novel therapeutics (Imhoff, 2016).

Several classes of chemically distinct metabolites from marine fungi have been reported in recent years which have a wide range of activities against different targets (Wu et al., 2015, 2016). From marine fungi alone, over thousand metabolites have been reported to have potential to be developed as drugs (Gomes et al., 2015), with several as anticancer compounds (please also see a detailed review, Bugni and Ireland, 2004 for historical inputs, taxonomy, ecological roles, distribution and chemistry as well biological activities of marine fungi), none of these have reached the market till now. However, for majority of these findings, complete taxonomy studies, biological targets and modes of interaction have not been identified yet. Due to these limitations, in this review, we cover anticancer compounds reported from marine fungi obtained from different sources such as deep-sea sediments, algae, sponge, mangrove endophytic and other marine fungi, discovered during 2012–2016 with a focus on summarizing the important findings and highlighting the lead compounds. Wherever explored, the biological targets and efficacies have been discussed as well. Novel anticancer compounds reported from marine fungi are given in Supplementary Table 1. They are arranged on the basis of sources the fungi isolated.

## Metabolites isolated from deep-sea sediment fungi

Deep-sea fungi inhabit at depths of thousand meters or below the surface (Swathi et al., 2013) where the sea environments are extreme; which are typically characterized by the absence of sunlight irradiation, predominantly low temperature, high hydrostatic pressure, and oligotrophy. Many reports indicate abundance and diversity of fungi in these environments (Hua et al., 2011; Mahé et al., 2013). Here, we present an account of metabolites reported from the deep-sea fungi during 2012–2016 that have displayed anticancer activities in various cell lines.

**Figure 1:**
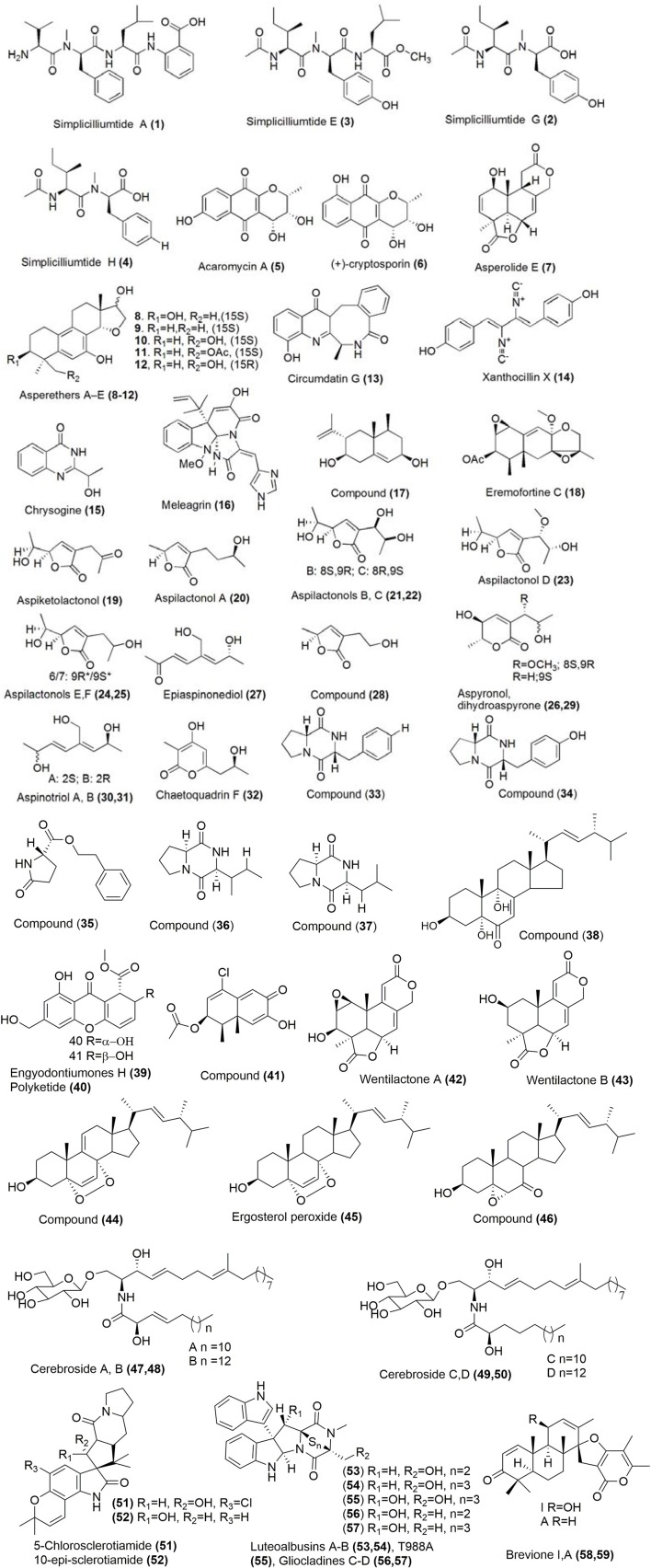
Structures of metabolites obtained from deep sea marine fungi. For complete chemical names, please see Supplementary Table 1.

Linear peptides, simplicilliumtides A, E, G, and H (**1–4**; Figure 1) were isolated from a culture broth of the deep-sea-derived fungal strain *Simplicillium obclavatum* EIODSF 020e collected in the East Indian Ocean. Simplicilliumtides A and G showed weak cytotoxicity toward human leukemia HL-60 cell line with IC_50_ values of 64.7 and 100 μM, and simplicilliumtides E and H showed weak cytotoxicity toward K562 cell line with IC_50_ values of 39.4 and 73.5 μM (Liang et al., 2016). Using a combination of fermentation and subsequent chromatographic separation, acaromycin A **(5)** and (+)-cryptosporin (**6**; Figure 1) were isolated from the deep-sea derived fungus *Acaromyces ingoldii* FS121 which was obtained from the South Sea in China. Using a combinaion of one and two-dimensional NMR as well as mass spectroscopic techniques, the chemical structures were elucidated and the absolute configuration was further determined by circular dichroism (CD) experiments. Compounds **(5)** and **(6)** exhibited considerable growth inhibition against tumor cell lines MCF-7, NCI-H460, SF-268, and HepG-2 with IC_50_ values <10 μM. The inhibitory effect of compound (**5**) against MCF-7 cell line was comparable to cytoxicity of cisplatin which was used as a positive control (Gao et al., 2016a). A new tetranorlabdane diterpenoid, asperolide E (**7**; Figure 1) was isolated from the deep sea sediment-derived fungus *Aspergillus wentii* SD-310. The cytotoxicity of compound **(7)** was evaluated against HeLa, MCF-7, and NCI-H446 cell lines which showed IC_50_ values of 10.0, 11.0, and 16.0 μM respectively (Li X.-D. et al., 2016).

Asperethers A-E (**8–12**; Figure 1), five new 20-nor-isopimarane diterpenoids having a 14,16-cyclic ether unit and a unique 6/6/6/5 tetracyclic skeleton, were discovered from the culture extract of *Aspergillus wentii* SD-310 from the deep-sea sediment sample. The chemical structure of these compounds was determined by mass spectrometry and NMR techniques (^1^H NMR, COSY, HSQC, HMBC) and the absolute configurations were supported by NOESY, X-ray crystallography, CD and computational methods. Compounds (**8–12**) displayed cytotoxic activities against A549 cell line with the IC_50_ values of 20, 16, 19, 17, and 20 μM, respectively, which were moderately higher than the positive control Adriamycin (Li X. et al., 2016).

Circumdatin G (**13**; Figure 1), was isolated from the culture of the deep-sea fungus *Aspergillus westerdijkiae* SCSIO 05233 which was isolated from a sediment sample in the South China Sea. Spectroscopic analysis using mass spectrometry and a variety of one and two-dimensional NMR techniques (DEPT, HMBC) led to the determination of its chemical structure. Compound (**13**) displayed weak antiproliferation activities toward K562 and promyelocytic HL-6 cell lines with IC_50_ values ranging between 25.8 and 44.9 μM (Fredimoses et al., 2015). Xanthocillin X **(14)**, chrysogine **(15)** and meleagrin (**16**; Figure 1) were discovered from *Penicillium commune* SD-118. The growth inhibition of compound **(14)** was evaluated against MCF-7, HepG2, NCI-H460, HeLa, DU145, and MDA-MB- 231 cell lines with the IC_50_ values of 12.0, 7.0, 10.0, 10.0, 8.0, and 8.0 μg/mL respectively. The cytotoxicity of compound **(15)** was moderate against SW1990 cell line with an IC_50_ value of 20.0 μg/mL, whereas compound **(16)** exhibited potent cytotoxicity against DU145 cell line with an IC_50_ value of 5.0 μg/mL. It also showed moderate cytotoxicity toward HepG2, NCIH460, HeLa, and MDA-MB-231 cell lines with IC_50_ values of 12.0, 22.0, 20.0, and 11.0 μg/mL respectively (Shang et al., 2012a; Zhao et al., 2012).

Using a combination of traditional and high-performance liquid chromatography techniques, eremophilane type sesquiterpene (**17**; Figure 1) and an analog of one tautomeric form of eremofortine C **(18)** were isolated from the Antarctic deep-sea fungus *Penicillium* sp. PR19 N-. The cytotoxicity studies of compounds **(17)** and **(18)** against HL-60 cells were evaluated which gave IC_50_ values of 45.8, 28.3 μM respectively. The inhibitory concentrations of compounds **(17)** and **(18)** against A-549 cells were found to have IC_50_ values of 82.8, 5.2 μM respectively (Lin et al., 2014). Nine new C_9_ polyketides named aspiketolactonol **(19)**, aspilactonols A–F (**20-25**; Figure 1), aspyronol **(26)** and epiaspinonediol **(27)** were isolated together with five known polyketides (*S*)-2-(2′-hydroxyethyl)-4-methyl-γ-butyrolactone **(28)**, dihydroaspyrone **(29)**, aspinotriol A **(30)**, aspinotriol B **(31)** and chaetoquadrin F **(32)** from *Aspergillus* sp. 16-02-1, which was collected from a deep-sea sediment at a Lau Basin hydrothermal vent in the southwest of the Pacific Ocean. NMR, mass and CD spectroscopy techniques were used to determine the chemical structure and assign the absolute configuration of these novel molecules. Compounds **(19-32)** exhibited significant cytotoxic activities with inhibitory rate (IR%) values at 100 μg/mL between 10 and 79% against human cancer cell lines K562, HL-60, HeLa, and BGC-823 (Chen X. et al., 2014). Six metabolites, (**33–38**; Figure 1) were isolated from a mutated deep-sea fungal strain of *Aspergillus versicolor* ZBY-3. Their inhibitory activities were determined aginst K562 cells at a concentration of 100 μg/mL which showed inhibitory rates of 54.6, 72.9, 23.5, 29.6, 30.9, and 51.1% respectively (Dong et al., 2014). From a deep-sea fungus *Engyodontium album* DFFSCS021, a new chromone engyodontiumones H **(39)** and a known polyketide (**40**; Figure 1) were isolated. Their cytotoxcities were determined which showed selectivity against human histiocytic lymphoma U937 cell line with the IC_50_values of 4.9 and 8.8 μM respectively (Yao et al., 2014).

An Antarctic deep-sea derived fungus *Penicillium* sp. PR19N-1 was the source of compound (**41**; Figure 1). Its chemical structures was established using IR, HRMS as well as one and two-dimensional NMR techniques. The cytotoxicity of compound **(41)** was modest against HL-60 and A549 cell lines with IC_50_ values of 11.8 and 12.2 μM respectively (Wu et al., 2013). The deep-sea sediment of the South China Sea was the source of fungus *Aspergillus dimorphicus* SD317 from which, Wentilactone A **(42)** and B (**43**; Figure 1) were isolated (Xu et al., 2015). Further exploration of induced apoptosis of Wentilactone A **(42)** displayed G2/M cell cycle arrest of human lung carcinoma cells (Lv et al., 2013). Wentilactone B **(43)** inhibited proliferation and migration of human hepatoma SMMC-7721 cells (Zhang et al., 2013). Seven secondary metabolites including ergosterol peroxide derivative **(44)**, ergosterol peroxide **(45)**, (22*E*,24*R*)-5α,6α-epoxy-3β-hydroxyergosta-22-ene-7-one **(46)**, and cerebroside A-D (**47-50**; Figure 1) were reported by Cui and coworkers in 2013 from the deep-sea fungus *Paecilomyces lilacinus* ZBY-1. These compounds exhibit cytotoxic activity against K562, MCF-7, HL-60, and BGC-823 cells with IC_50_ of 22.3–139.0 μM (Cui et al., 2013). The compounds 5-chlorosclerotiamide **(51)** and 10-epi-sclerotiamide **(52)** were the secondary metabolites of the deep-sea fungus *Aspergillus westerdijkiae* DFFSCS013, which showed excellent cytotoxicity against K562 cell line with IC_50_ of 44 and 53 μM respectively (Peng et al., 2013). Another deep-sea sediment in the South China Sea was the source of fungus *Acrostalagmus luteoalbus* SCSIO F457 from which metabolites luteoalbusins A–B **(53, 54)**, T988A **(55)** and gliocladines C–D **(56, 57)** were isolated (Figure 1). The cytotoxicity of compound **(53)** was evaluated against SF-268 MCF-7 NCI-H460 HepG-2 cell lines, which gave IC_50_ values of 0.46, 0.233, 1.15, and 0.91 μM respectively. Similarly, the cytotoxicity of compound **(54)** against SF-268 MCF-7 NCI-H460 HepG-2 gave IC_50_ values of 0.59, 0.25, 1.31, 1.29 μM respectively. Compounds **(55–57)** were slightly less cytotoxic against SF-268, MCF-7, NCI-H460, and HepG-2 with IC_50_ values ranging in between 0.91 and 17, 7 μM respectively. The positive control cisplatin, exhibited cytotoxicity against SF-268, MCF-7, NCI-H460, and HepG-2 cell lines with IC_50_ values of 4.7, 3.9, 2.9, and 2.4 μM respectively (Wang et al., 2012). Breviones I and A **(58, 59)** were isolated from the deep-sea fungus *Penicillium* sp. in China (Figure 1). The cytotoxicity of compounds **(58)** and **(59)** were determined against MCF-7 cells lines, which gave IC_50_ values of 7.44 and 28.4 μM respectively. The IC_50_ of compound **(58)** against A549 cells was found to be 32.5 μM (cisplatin, the positive control, gave IC_50_ values of 8.0 and 8.9 μM against these two tumor cell lines; Li et al., 2012).

## Compounds from algae-associated fungi

**Figure 2:**
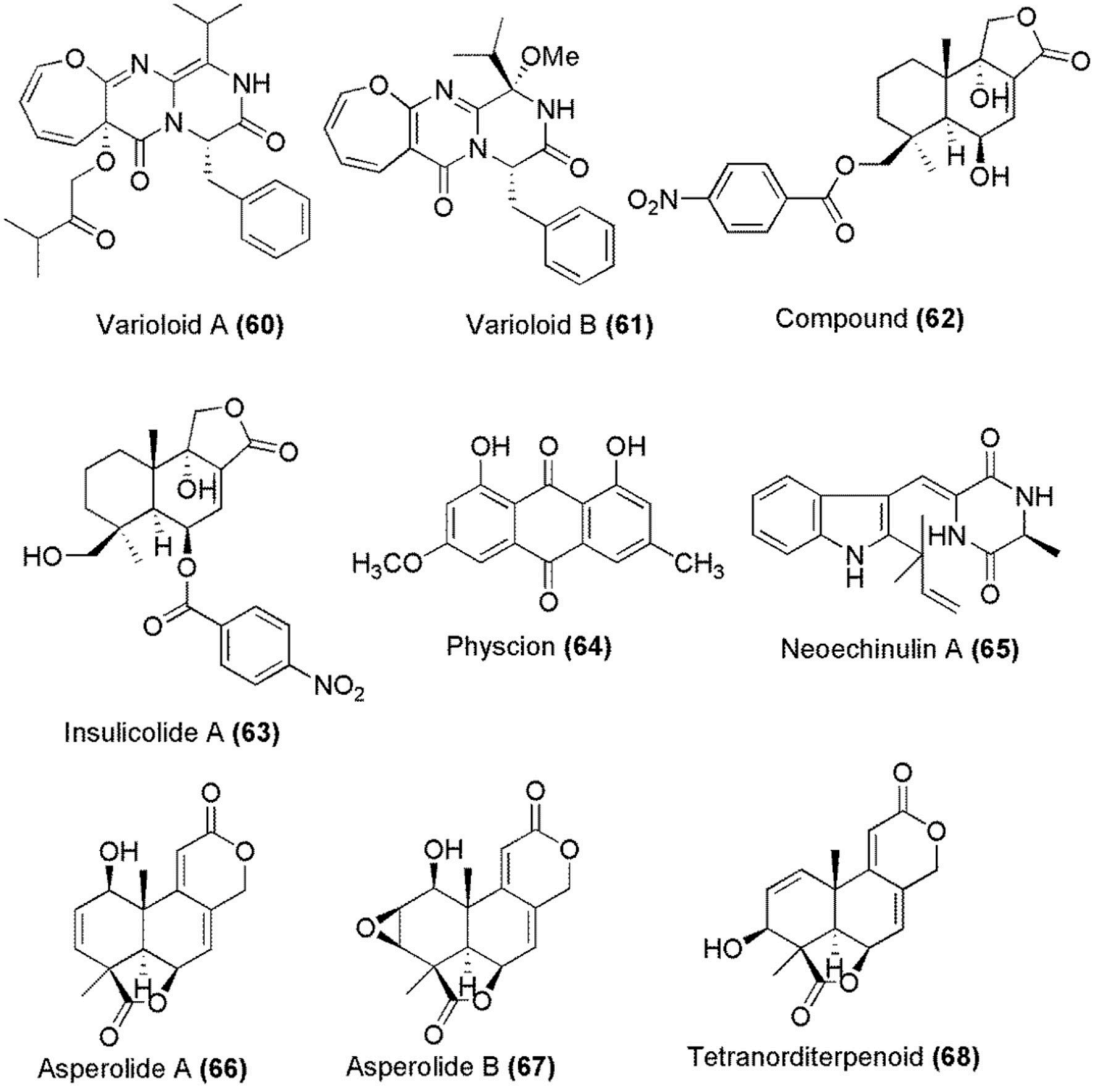
Structures of metabolites obtained from marine algae associated fungi.

Marine algae-derived endophytic fungus *Paecilomyces variotii* EN-291 was the source of indole derivatives varioloid A **(60)** and varioloid B (**61**; Figure 2). Both compounds were cytotoxic against A549, HCT116, and HepG2 cell lines with IC_50_ values between 2.6 and 8.2 μg/mL (Zhang et al., 2016). Another marine algae-derived fungus *Aspergillus ochraceus* Jcma1F17 gave cinnamolide derivative **(62)** and a known compound insulicolide A **(63)** whose chemical structures are given in Figure 2. The cytotoxicity of these compounds was determined against H1975, U937, K562,−823, Molt-4,−7, A549, HeLa, HL60, and Huh-7 human cancer cell lines, which gave IC_50_ values between 1.95 and 6.35 μM (Fang et al., 2014).

Marine red alga *Lomentaria catenata* was collected at Guryongpo, NamGu, PoHang in Republic of Korea. From the surface of this alga, fungus *Microsporum* sp. (MFS-YL) was obtained from which physcion **(64)** was isolated (Figure 2). Physcion **(64)** induced apoptosis in HeLa cells and its effect on the expressions of p53, p21, Bax, Bcl-2, caspase-9, and caspase-3 proteins were investigated. The western blot analysis revealed that physcion **(64)** induces cell apoptosis through down-regulation of Bcl-2 expression, up-regulation of Bax expression, and activation the caspase-3 pathway. Additionally, physcion **(64)** also induced the formation of reactive oxygen species (ROS) in HeLa cells (Wijesekara et al., 2014). A prenylated indole alkaloid neoechinulin A **(65)** (Figure 2) was obtained from a marine-derived fungus, *Microsporum* sp. (MFS-YL), which was isolated from the surface of a marine red alga *Lomentaria catenata*, collected at Guryongpo, NamGu, PoHang in the Republic of Korea. Neoechinulin A **(65)** has shown the cytotoxic effect on human cervical carcinoma HeLa cells and its apoptosis induction in HeLa cells was investigated by the expressions of p53, p21, Bax, Bcl-2, Caspase 9, and Caspase 3 proteins. Western blot analysis revealed that neoechinulin A **(65)** could induce cell apoptosis through down-regulation of Bcl-2 expression, up-regulation of Bax expression and activation the caspase-3 pathway (Wijesekara et al., 2013). Marine alga, *Sargassum* sp. was the source of endophytic fungus *Aspergillus wentii* EN-48 from which asperolides A–B **(66–67)** together with tetranorlabdane diterpenoid derivative **(68)**, wentilactones A **(42)** and B **(43)** were isolated. The cytotoxicity of these compounds **(66–68, 42, 43)** was moderate against HeLa, HepG2, MCF-7, MDA-MB-231, NCI-H460, SMMC-7721 and SW1990 tumor cell lines. Wentilactone B **(43)** was the most potent among the tested compounds (IC_50_ = 17 μM) (Sun H.-F. et al., 2012).

## Compounds from mangrove endophytic fungi

**Figure 3:**
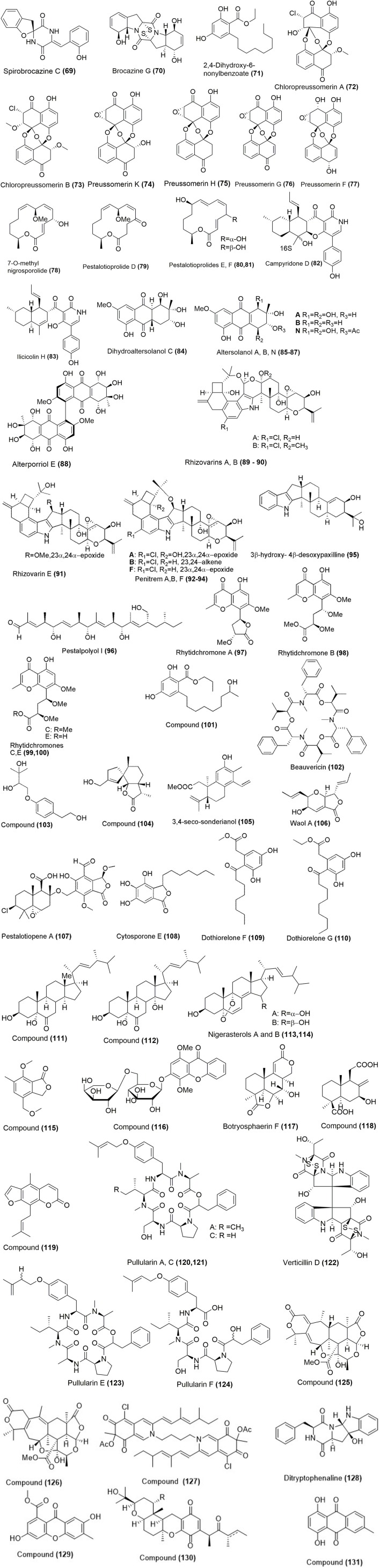
Chemical structures of metabolites associated with marine mangrove endophytic fungi. For complete chemical names, please see Supplementary Table 1.

Using one strain many compounds (OSMAC) approach, spirobrocazines C **(69)** and brocazine G **(70)** were obtained from mangrove-derived fungus *Penicillium brocae* MA-231 (Figure 3). Their chemical structures and absolute stereochemical configurations were determined by spectroscopic analysis, computational calculations and X-ray diffraction. Spirobrocazine C **(69)** showed moderate activity against A2780 cells (IC_50_ 59 μM) while compound **(70)** showed strong activity against A2780 and A2780 CisR cell with the IC_50_ values of 664 and 661 nM respectively, which were much better than that of the positive control cisplatin, which gave IC_50_ values of 1.67 and 12.63 μM respectively (Meng et al., 2016). 2,4-Dihydroxy-6-nonylbenzoate (**71**; Figure 3) was isolated from a mangrove endophytic fungus, *Lasiodiplodia* sp. 318, which was collected from *Excoecaria agallocha* in Mangrove National Nature Reserve in Gaoqiao, Zhanjiang city, Guangdong Province, China. Its structure was established by spectroscopic techniques (one and two-dimensional NMR, HR-ESI-MS), and electronic CD experiment. Compound **(71)** exhibited cytotoxicity against MMQ and GH3 cell lines with the IC_50_ values of 5.2 and 13.0 μM respectively (Huang et al., 2017). Endophytic fungus, *Lasiodiplodia theobromae* ZJ-HQ1 was isolated from a healthy leaf of the marine mangrove *A. ilicifolius*, which was collected from Zhanjiang Mangrove Nature Reserve in Guangdong Province, China. This fungus gave two new chlorinated preussomerins, chloropreussomerins A and B **(72, 73)** together with spreussomerin K **(74)**, preussomerin H **(75)**, preussomerin G **(76)**, and preussomerin F **(77)** as their metabolites (Figure 3). Their chemical structures were elucidated by a combination of spectroscopic techniques. The absolute configurations of **(72)** and **(73)** were determined by single-crystal X-ray diffraction techniques. Compounds **(72)** and **(73)** were the first chlorinated compounds in the preussomerins family, which showed potent *in vitro* cytotoxicity against A549 and MCF-7 human cancer cell lines with IC_50_ values ranging from 5.9 to 8.9 μM. Compounds **(74–77)** exhibited significant bioactivity against A549, HepG2, and MCF-7 human cancer cell lines with the IC_50_ values of 2.5–9.4 μM (Chen et al., 2016).

7-O-methylnigrosporolide **(78)**, pestalotioprolides D-F **(79–81)** were isolated from mangrove derived endophytic fungus *Pestalotiopsis microspore* (Figure 3), which was obtained from fresh healthy fruits of *Drepanocarpus lunatus* (Fabaceae) collected from Douala, Cameroon. Co-culture of *P. microspora* with *Streptomyces lividans* resulted in roughly ten-fold enhancement in the production accumulation of compounds **(80)** and **(81)** compared to axenic fungal control. Their chemical structures were determined by the analysis of NMR and mass spectroscopy data. The cytotoxicity of these compounds **(78-81)** was significant against murine lymphoma cell line L5178Y, which showed IC_50_ values of 0.7, 5.6, 3.4, and 3.9 μM respectively. Compound **(80)** was potent against the human ovarian cancer cell line A2780 with an IC_50_ value of 1.2 μM (Liu et al., 2016). Campyridones D **(82)** and ilicicolin H **(83)** were isolated from *Campylocarpon* sp. HDN13-307 (Figure 3), which was obtained from the root of mangrove plant *Sonneratia caseolaris*. Their chemical structures and absolute configurations were determined on the basis of spectroscopic analysis and electronic CD results. Campyridone D **(82)** and ilicicolin H **(83)** were cytotoxic against HeLa cell with the IC_50_ values of 8.8 and 4.7 μM respectively (Zhu et al., 2016).

Dihydroaltersolanol C **(84)**, altersolanols A, B, N **(85-87)**, and alterporriol E **(88)** were isolated from white bean solid culture media of the endophytic fungus, *Stemphylium globuliferum*, collected from the Egyptian mangrove plant *Avicennia marina* (Figure 3). Their structures were elucidated using one and two-dimensional NMR spectroscopy as well as high-resolution mass spectroscopy (Moussa et al., 2016). Dihydroaltersolanol C **(84)**, altersolanol A **(85)**, B **(86)**, and alterporriol E **(88)** exhibited toxicity against L5178Y mouse lymphoma cell line with the IC_50_ values of 3.4, 2.5, 3.7, and 6.9 μM (Liu Y. et al., 2015). Altersolanol N **(87)** also exhibited potent cytotoxicity against L5178Y mouse lymphoma cell line with IC_50_ values in the low micromolar range (Debbab et al., 2012). Altersolanol A **(85)** showed cytotoxic activity against 34 human cancer cell lines *in vitro*, with mean IC_50_ (IC_70_) values of 0.005 μg/mL (0.024 μg/ml) respectively (Mishra et al., 2015). The cellular activity of altersolanol A **(85)** has been studied in detail, which has shown that it is a kinase inhibitor which induces cell death by apoptosis through caspase dependent pathway. Altersolanol A **(85)** inhibited a variety of kinases, which suggested that the kinase inhibition might be the mechanism for the cytotoxic activity (Debbab et al., 2009). Further studies revealed that its antitumor potential was linked to pro-apoptotic and anti-invasive activity that occurred through the inhibition of NF-κB transcriptional activity (Teiten et al., 2013). *Mucor irregularis* QEN-189, an endophytic fungus obtained from the fresh inner tissue of the marine mangrove plant *Rhizophora stylosa*, collected in Hainan Island, China was the source of rhizovarins A, B, E **(89–91)**, penitrems A, C, F **(92–94)**, and 3β-hydroxy- 4β-desoxypaxilline **(95)** whose chemical structures are shown in Figure 3. The structures of these compounds were determined by detailed spectroscopic analysis. Compounds **(89-94)** were cytotoxic against the human A-549 cell lines with IC_50_ values of 11.5, 6.3, 9.2, 8.4, 8.0, 8.2, and 4.6 μM, while compounds **(89, 90, 92–95)** showed cytotoxicity against the human HL-60 cell lines with IC_50_ values of 9.6, 5.0,7.0, 4.7, 3.3, and 2.6 μM respectively. Adriamycin, a positive control, exhibited cytotoxicity with the IC_50_ values of 0.30 and 0.06 μM against A-549 and HL-60 cell lines respectively (Gao et al., 2016b).

Endophytic fungus *Pestalotiopsis clavispora* isolated from the mangrove plant *Rhizophora harrisonii* was the source of a new polyketide derivative pestalpolyol I (**96**; Figure 3). The chemical structure of the new compound was determined using one and two-dimensional NMR spectroscopy, as well as by high-resolution mass spectrometry. Compound **(96)** displayed cytotoxicity against the mouse lymphoma cell line L5178Y activity with an IC_50_ value of 4.10 μM (Perez et al., 2016). Four highly oxygenated chromones, rhytidchromone A, B, C, and E **(97–100)** were isolated from the culture broth of a mangrove-derived endophytic fungus, *Rhytidhysteron rufulum*, which was obtained from Thai *Bruguiera gymnorrhiza* (Figure 3). Their structures were determined by analysis of 1D and 2D NMR spectroscopic data. The structure of rhytidchromone A **(97)** was further confirmed by single-crystal X-ray diffraction analysis. Compounds **(97–100)** displayed cytotoxicity against Kato-3 cell lines with the IC_50_ values ranging from 16.0 to 23.3 μM, while rhytidchromones A and C were active against MCF-7 cells with the IC_50_ values of 19.3 and 17.7 μM respectively (Chokpaiboon et al., 2016).

Another compound ethyl-2,4-dihydroxy-6-(8′-hydroxynonyl)- benzoate (**101**; Figure 3) was isolated from a mangrove endophytic fungus, *Lasiodiplodia* sp. 318# and its complete chemical structure was elucidated by spectroscopic techniques. The compound **(101)** was cytotoxic against several cell lines with the IC_50_ values of 10.1 μM (MDA-MB-435), 12.5 μM (HepG2), 11.9 μM (HCT-116), 13.31 μM (A549), and 39.74 μM (THP1) respectively (Li J. et al., 2016). Mangrove derived endophytic fungus *Fusarium* sp. (No. DZ27) in the South China Sea was the source of beauvericin **(102)**, a cyclic peptide (Figure 3), and its chemical structure was deduced by spectroscopic methods and also using the reference data from the literature. Beauvericin **(102)** was potent in the growth inhibition of KB and KBv200 cells with the IC_50_ values of 5.76 and 5.34 μM. Further analysis of beauvericin **(102)** activity was done, which showed that it induced apoptosis through the decrease of reactive oxygen species generation, loss of mitochondrial membrane potential, release of cytochrome C, activation of Caspase-9 and -3, and cleavage of PARP and did not regulate Bcl-2 or Bax expression (Tao et al., 2015).

Mangrove associated endophytic fungus *Penicillium* sp.FJ-1 of *Avicennia marina*, which was collected in Fujian, China was the source of two new metabolites; compounds **(103)** and **(104)** as shown in Figure 3. Their chemical structures were determined using NMR and mass spectroscopy. The antiproliferative activity of compound **(103)** was weak against Tca8113 and MG-63 cells with the IC_50_ values of 26 and 35 μM respectively. The positive control, taxol, gave the IC_50_ values of 46 and 10 nM with Tca8113 and MG-63 cell lines respectively. The IC_50_ value of compound **(104)** on Tca8113 and normal liver cell line WRL-68 was 10 and 58 μM respectively. Compound **(104)** also showed anti-tumor effect on MG-63 cells with an IC_50_ value of 55 nM. Compound **(104)** was also tested against nude mice, which showed significant inhibition of tumor growth of human osteosarcoma (Zheng et al., 2014). A known diterpenoid 3,4-seco-sonderianol (**105**; Figure 3) was isolated from endophytic fungus J3 of *Ceriops tagal* collected in the mangrove reserve of Dong Zhai Gang, Hainan province, China. Its structure was elucidated using spectroscopic methods including 1D and 2D NMR (HMQC, ^1^H-^1^H COSY and HMBC). Compound **(105)** exhibited activities against K562, SGC-7901, and BEL-7402 cell lines with the IC_50_ values of 9.2, 15.7, and 25.4 μg/mL respectively. Paclitaxel was used as the positive control, which displayed the IC_50_ values of 5.1 μg/mL against K562, 1.6 μg/mL against SGC-7901 and 6.3 μg/mL against BEL-7402 cel lines respectively (Zeng et al., 2015). Waol A **(106)**, pestalotiopene A **(107)**, cytosporone E **(108)** were obtained from the endophytic fungus *Acremonium strictum*, isolated from the mangrove tree *Rhizophora apiculata* (Figure 3). The chemical structures of the isolated compounds were elucidated on the basis of comprehensive NMR and mass spectrometry analysis. Compounds **(106–108)** showed moderate cytotoxic activity against human cisplatin-sensitive (IC_50_ values 27.1, 76.2, and 8.3 μM respectively) and resistant A2780 cell lines (IC_50_ values 12.6, 30.1, and 19.0 μM respectively) (Hammerschmidt et al., 2014).

Mangrove endophytic fungus *Dothiorella* sp., which was obtained from the bark of the mangrove tree *Aegiceras corniculatum* at the estuary of Jiulong River, Fujian Province of China, was the source of two new polyketides, named dothiorelones F **(109)** and G **(110)** as shown in Figure 3. Their chemical structures were determined on the basis of NMR data and mass spectrometry. Dothiorelones F **(109)** and G **(110)** showed significant cytotoxicity against Raji cancer cell line with an IC_50_ value of 2 μg/mL (Du and Su, 2014). The mangrove endophytic fungus *Aspergillus terreus* (No. GX7-3B), which was obtained from a branch of *Bruguiera gymnoihiza* (Linn.) growing on the coastal salt marsh of the South China Sea was the source of compounds **(111**, **112)** and beauvericin **(102)** as shown in Figure 3. Their chemical structures were determined by the analysis of the spectroscopic data. The cytotoxicity of compounds **(111)** and **(102)** ranged from moderate to strong against MCF-7, A549, HeLa, and KB cell lines with the IC_50_ values of 4.98 and 2.02 (MCF-7), 1.95 and 0.82 (A549), 0.68 and 1.14 (HeLa) and 1.50 and 1.10 μM (KB) respectively. The inhibitory activity of compound **(112)** was weak against these tumor cell lines (Deng C. M. et al., 2013). Endophytic fungus *Aspergillus niger* MA-132 was isolated from mangrove plant *Avicennia marina*, which was the source of two sterol derivatives nigerasterols A and B (**113, 114)** as shown in Figure 3. The chemical structures and absolute configurations of these compounds were determined using spectroscopic methods. Modified version of Mosher's method was used to confirm the absolute configuration of compound **(113)**. Nigerasterols A and B **(113, 114)**, which represent the first 5,9-epidioxy-sterol compounds of marine origin were evaluated for cytotoxicity. Nigerasterol B **(114)** displayed potent activity against the tumor cell line HL60 with an IC_50_ value of 1.50 μM, while nigerasterol A **(113)** displayed stronger activity with an IC_50_ value of 0.30 μM. Both compounds **(113)** and **(114)** displayed potent activities against A549 cell line with the IC_50_ values of 1.82 and 5.41 μM respectively (Liu et al., 2013).

A new isobenzofuranone, 4-(methoxymethyl)-7-methoxy-6-methyl-1(3H)-isobenzofuranone (**115**; Figure 3) was isolated from the mangrove endophytic fungus *Penicillium* sp. ZH58, which was obtained from the South China Sea coast. Its chemical structure was determined by the analysis of spectroscopic data. Compound **(115)** exhibited cytotoxicity against KB and KB_V200_ cells with the IC_50_ values of 6 and 10 μg/mL, respectively (Yang et al., 2013). A new xanthone derivative (**116**; Figure 3) was isolated from the culture of mangrove endophytic fungus, *Phomopsis* sp. (ZH76). Its chemical structure was determined on the basis of spectroscopic data. Compound **(116)** inhibited the growth of HEp-2 and HepG2 cells with the IC_50_ values of 9 and 16 μM respectively (Huang et al., 2013). Mangrove fungus *Aspergillus terreus* (No. GX7-3B) led to the production of two metabolites: compound **(117)** and compound **(118)** as shown in Figure 3. The chemcial structures of these compounds were determined on the basis of spectroscopic data. Compound **(117)** showed inhibitory activity toward MCF-7 and HL-60 cancer cell lines with the IC_50_ values of 4.4 and 3.4 μM, respectively. The cytotoxicity of compound **(118)** was promising against HL-60 cell line with an IC_50_ value of 0.6 μM (Deng C. et al., 2013). Mangrove endophytic fungus, *Penicillium* sp. ZH16 was obtained from the South China Sea, which produced furanocoumarin derivative **(119)** as shown in Figure 3. Its chemical structure was determined by the analysis of NMR and mass spectroscopic data. Compound **(119)** was cytotoxic against KB and KB_V_200 cells with the IC_50_ values 5 and 10 μg/mL respectively (Huang Z. et al., 2012). Endophytic fungus *Bionectria ochroleuca*, which was obtained from the inner leaf tissues of the plant *Sonneratia caseolaris* in Hainan island (China) produced pullularin A **(120)**, pullularin C **(121)**, verticillin D **(122)** and pullularins E and F **(123, 124)** as shown in Figure 3. Their chemical structures were established using NMR spectroscopy and high-resolution mass spectrometry. Compounds **(120–124)** were cytotoxic against the mouse lymphoma cells (L5178Y) with the EC_50_ values between 0.1 and 6.7 μg/mL (Ebrahim et al., 2012).

Meroterpenes **(125–127)** were isolated from the marine fungus *Penicillium* sp. 303 cultured from sea water samples obtained from Zhanjiang Mangrove National Nature Reserve in Guangdong Province, China (Figure 3). The isolated compounds are structurally related to the miniolutelide class of meroterpenoids and were identified as derivatives of miniolutelide B. Compounds **(125)** and **(126)** showed moderate cytotoxic activities against a panel of cancer cell lines including MDA-MB-435, HepG2, HCT-116 and A549 cell lines. Compound **(127)** showed potent cytotoxic activity with IC_50_ values of 7.13 μM against MDA-MB-435 (Li J. et al., 2014). Ditryptophenaline **(128)** was isolated from mangrove endophytic fungus No·Gx-3a in the South China Sea (Figure 3). Ditryptophenaline showed strong inhibitory activity on KB and KBv200 cell lines with LD_50_ values of 8.0 and 12.0 μM (Yang et al., 2013b). A marine fungus *Phomopsis* sp. (No. SK7RN3G1) was obtained from mangrove sediment of Shankou in Hainan, China, which led to the production of a new xanthone derivative **(129)** as shown in Figure 3. Its chemical structure was determined by spectroscopic methods and it was found to be cytotoxic against HEp-2 and HepG2 cells with the IC_50_ values of 8 and 9 μg/mL (Yang et al., 2013c). The endophytic fungus *Nigrospora* sp. MA75 was obtained from the marine semi-mangrove plant *Pongamia pinnata* that led to the production of a new quinone derivative (**130**; Figure 3) which was isolated from *Nigrospora* sp. MA75, an. The chemical structure of compound **(130)** was elucidated by detailed spectroscopic analysis and absolute configuration determination. Compound **(130)** showed potent inhibition growth of MCF-7, SW1990, and SMMC7721 tumor cell lines with the IC_50_ values of 4, 5, and 7 μg/mL respectively (Shang et al., 2012b). Anthracene derivative **(131)** was isolated from mangrove endophytic fungus No.5094 which was collected in the South China Sea as shown in Figure 3. The compound was identified on the basis of spectral analysis. Compound **(131)** showed strong inhibitory activity with KB and KBv200 cell lines having the LD_50_ values of 5.5 and 10.2 μM respectively (Yang et al., 2013a).

## Compounds from marine sediment-derived fungi

**Figure 4:**
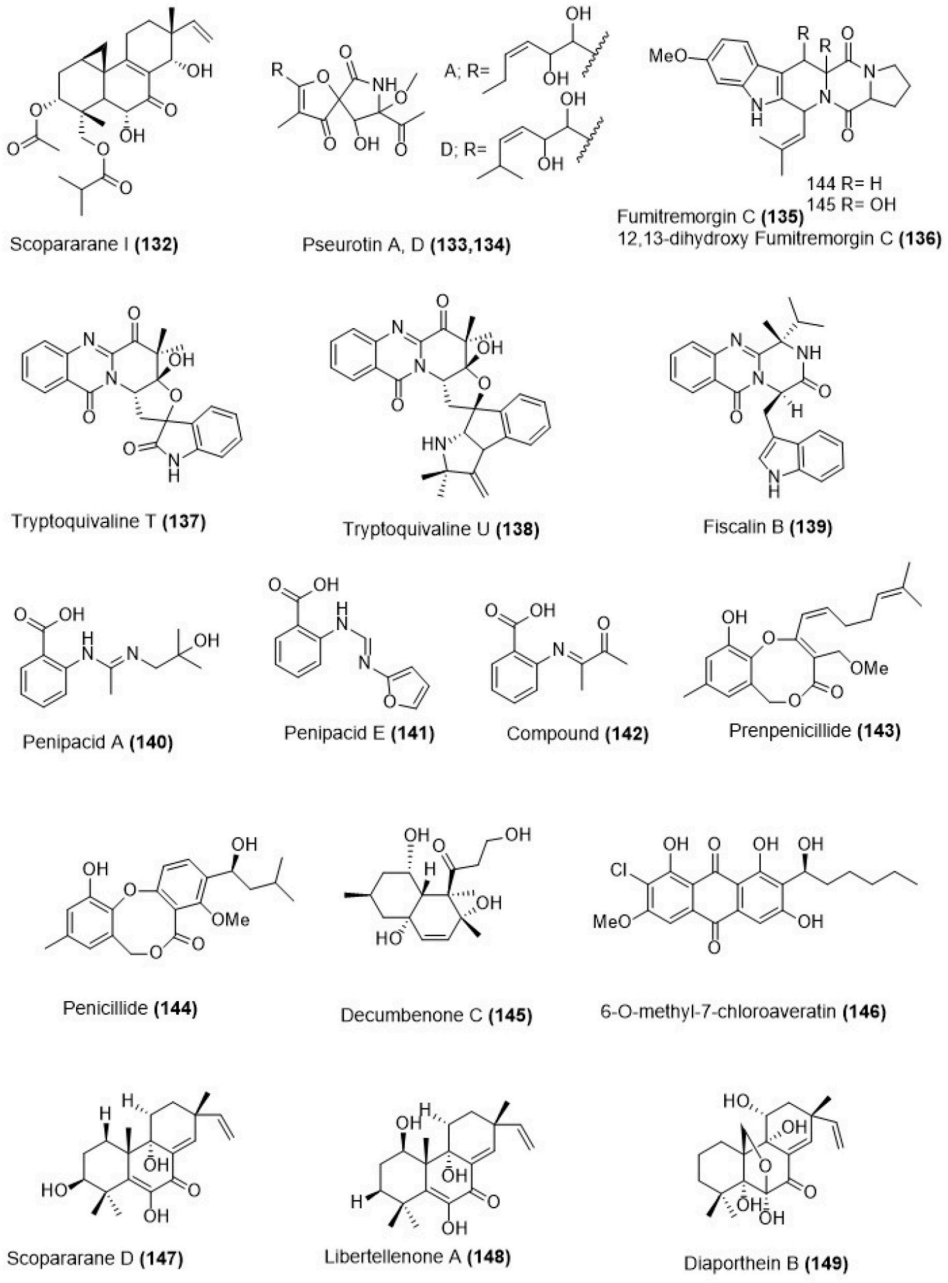
Chemical structures of metabolites obtained from marine sediment derived fungi. For complete chemical names, please see Supplementary Table 1.

Marine sediment-derived fungus *Eutypella* sp. FS46 was obtained from the South China Sea. The culture of this fungus produced a pimarane-type diterpene, scopararane I **(134)** as shown in Figure 4. Compound (**132**) showed moderate cytotoxicity against MCF-7, NCI-H460 and SF-268 cell lines with the IC_50_ values 83.9, 13.5, and 25.3 μg/ mL respectively (Liu et al., 2017). Hetero-spirocyclic γ-lactams pseurotin A **(133)**, pseurotin D **(134)**, alkaloids fumitremorgin C **(135)**, and 12,13-dihydroxy fumitremorgin C (**136**; Figure 4) were isolated from *Aspergillus* sp. (BRF 030) which was obtained from the sediments collected on the northeast coast of Brazil. Pseurotin A **(133)**, pseurotin D **(134)**, fumitremorgin C **(135)**, and 12,13-dihydroxy-fumitremorgin C **(136)** showed toxicity against HCT-116 cell line with the IC_50_ values 72.0, 85.0, 15.1, and 4.5 μM (Saraiva et al., 2015). Tryptoquivaline T **(137)**, tryptoquivaline U **(138)**, and fiscalin B **(139)** were isolated from *Neosartorya fischeri* which was obtained from marine mud in the intertidal zone of Hainan Province of China (Figure 4). The bioactivity of compounds (**137–139)** toward apoptosis of HL-60 cells were done which showed the IC_50_ values of 82.3, 90.0, and 8.8 μM respectively (Wu et al., 2015). Fungus *Penicillium paneum* SD-44 was obtained from marine sediment sample in the South China Sea which produced anthranilic acid derivatives penipacids A and E (**140, 141**; Figure 4) together with one known analog **(142)**. Their chemical structures were deduced using NMR and mass spectrometry analysis. Penipacids A **(140)** and E **(141)** inhibited RKO cell growth with the IC_50_ values of 8.4 and 9.7 μM while compound **(142)** was cytotoxic against HeLa cell line with an IC_50_ value of 6.6 μM, which was better than the positive control fluorouracil (IC_50_ = 25.0 and 14.5 μM against RKO and HeLa cells lines respectively; Li et al., 2013).

Marine-derived fungus *Penicillium* sp. ZLN29 was obtained from the sediments collected in the Jiaozhou Bay of China from which penicillide derivative **(143)** and a known polyketide compound **(144)** were isolated (Figure 4). Compounds **(143)** and **(144)** showed weak cytotoxicity against HepG2 cell line with the IC_50_ values of 9.9 and 9.7 μM respectively (Gao et al., 2013a). The marine fungus *Aspergillus sulphureus* KMM 4640 was obtained from marine sediments which produced a new decalin derivative decumbenone C **(145)** as shown in Figure 4. Decumbenone C **(145)** was cytotoxic against SK-MEL-5 human melanoma cells with an IC_50_ value of 0.9 μM (Zhuravleva et al., 2012). Marine-derived fungus *Aspergillus* sp. SCSIO F063 was obtained from a marine sediment sample, collected in the South China Sea which produced chloroaveratin derivative **(146)** as shown in Figure 4. Chemical structure determination was done by spectroscopic analyses that included mass spectrometry and NMR. Compound **(146)** showed inhibitory activity against three human tumor cell lines; SF-268, MCF-7, and NCI-H460 with the IC_50_ values of 7.1, 6.6, and 7.4 μM respectively (Huang H. et al., 2012).

Marine-derived fungus *Eutypella scoparia* FS26 that had been obtained from the sediment collected in the South China Sea produced scopararane D **(147)**, libertellenone A **(148)** and diaporthein B **(149)** whose structures are depicted in Figure 4. All isolated compounds were assessed for their antiproliferative activity using a cytotoxicity (MTT) assay against three different human cancer cell lines: MCF-7 (breast), NCI-H460 (lung), and SF-268 (brain). Compound **(147)** showed only mild antiproliferative activity with the IC_50_ values between 25.6 and 46.0 μM, whereas, libertellenone A **(148)** and diaporthein B **(149)**, revealed potent antiproliferative activities with IC_50_ values ranging from 4.4 to 20.0 μM, compared to cisplatin (IC_50_ = 1.5–9.2 μM) (Sun L. et al., 2012).

## Compounds from sponge associated fungi

**Figure 5:**
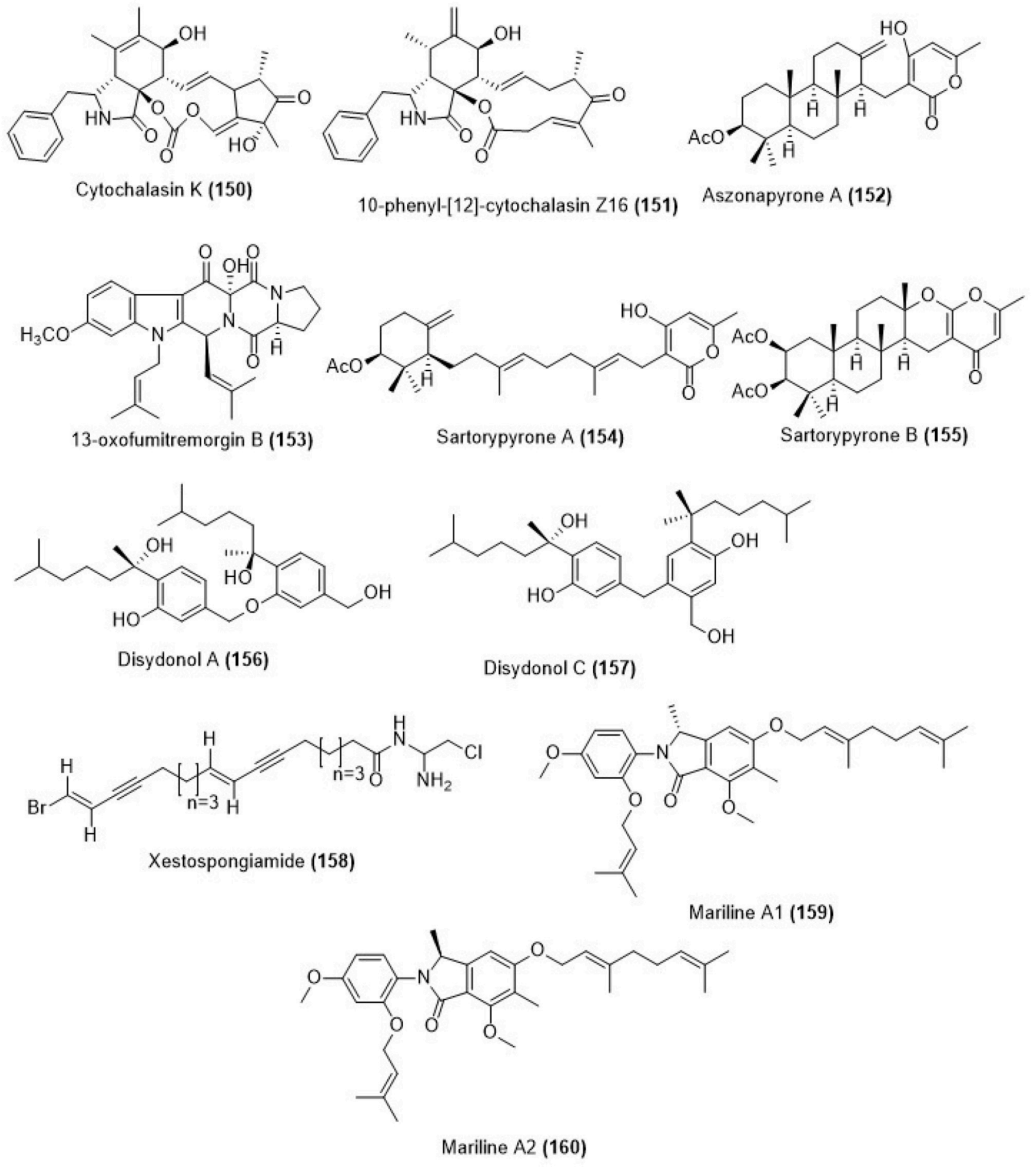
Chemical structures of metabolites obtained from marine sponge associated fungi.

Fungus *Arthrinium arundinis* ZSDS1-F3 was collected from sponge *Phakellia fusca* in Xisha Islands of China which led to the isolation of metabolites cytochalasin K **(150)** and compound **(151)** as shown in Figure 5. Compounds **(150)** and **(151)** showed cytotoxicity against K562, A549, Huh-7, H1975, HL60, HeLa, and MOLT-4 cell lines with the IC_50_ values ranging between 1.1 and 47.4 μM. Compound **(150)** showed cytotoxicity against K562, A549, Huh-7, H1975, MCF-7, U937, BGC823, HL60, HeLa, and MOLT-4 cell lines, with IC_50_ values of 10.5, 13.7, 10.9, 19.1, 11.1, 47.4, and 11.8 μM respectively while compound **(151)** was cytotoxic against K562, A549, Huh-7, H1975, MCF-7, U937, BGC823, HL60, HeLa MOLT-4 cell lines with IC_50_ values of 6.2, 1.1, >50, 14.2, 18.5, 3.4, 18.8, 6.2, 3.2, and 4.1 μM respectively. The positive control trichostatin A was cytotoxic to the same cell lines with the IC_50_ values of 0.24, 0.05, 0.09, 0.10, 0.08, 0.06, 0.09, 0.09, 0.11, and 0.03 μM respectively (Wang et al., 2015). Coral-derived fungus *Neosartorya laciniosa* (KUFC 7896) which was collected from the coastal forest soil at Samaersarn island, Chonburi Province, Thailand was the source of aszonapyrone A **(152)**, 13-oxofumitremorgin B **(153)**, sartorypyrone A **(154)** and sartorypyrone B **(155)** as shown in Figure 5. The chemical structures of the new compounds were determined on the basis of one and two-dimensional NMR spectral analysis as well as HR-ESIMS. Aszonapyrone A **(152)**, 13-oxofumitremorgin B **(153)**, sartorypyrone A **(154)** and sartorypyrone B **(155)** were evaluated for their ability to inhibit the growth of MCF-7, NCI-H460, and A375-C5 cell lines. The cytotoxicity results displayed that, among the meroditerpenes tested, aszonapyrone A **(152)** was the most potent compound showing strong growth inhibitory activity with GI_50_ = 13.6, 11.6 and 10.2 μM for MCF- 7, NCI-H460 and A375-C5 cell lines respectively. Sartorypyrone B **(155)** was also potent in growth inhibition, however, it was less active than aszonapyrone A **(154)** having the GI_50_ values 17.8, 20.5, and 25.0 μM for MCF-7, NCI-H460 and A375- C5 cell lines respectively. Another compound 13-oxofumitremorgin B **(153)** exhibited only weak inhibitory activity against all the three cell lines (GI_50_ = 115.0, 123.3, and 68.6 μM for MCF-7, NCI-H460 and A375-C5 cell lines respectively; Eamvijarn et al., 2013).

The marine-derived fungus *Aspergillus* sp., which was obtained from the sponge *Xestospongia testudinaria*, was collected from the South China Sea that gave two phenolic bisabolane sesquiterpenoid dimers, disydonols A and C **(156, 157**) as shown in Figure 5. Their chemical structures were determined on the basis of spectroscopic analysis. Compound **(156)** exhibited *in vitro* moderate cytotoxicity toward HepG-2 and Caski human tumor cell lines with the IC_50_ values of 9.31 and 12.40 μg/mL respectively. Compound **(157)** also displayed selectivity against HepG-2 and Caski human tumor cell lines with the IC_50_ values of 2.91 and 10.20 μg/mL respectively (Sun L.-L. et al., 2012). A new polyacetylene, xestospongiamide **(158)** was obtained from the Red Sea sponge, *Xestospongia* sp. which was collected from deep waters of Sharm Obhur, Jeddah, Saudi Arabia (Figure 5). Compound **(158)** showed antitumor effect against both Ehrlich ascites carcinoma and lymphocytic leukemia (LD_50_ 5.0 μM each) (Ayyad et al., 2015).

A marine-derived fungus of the genus *Stachylidium* was isolated from the sponge *Callyspongia* cf. *C. flammea*. Chemical investigation of the bioactive fungal extract led to the isolation of the novel phthalimidine derivatives marilines A1 and A2 **(159, 160)** whose chemical structures are shown in Figure 5. The absolute configurations of the enantiomeric compounds **(159)** and **(160)** were assigned using a combination of experimental circular dichroism (CD) investigation and quantum chemical CD calculations. The skeleton of marilines is unusual and its biosynthesis was suggested to require uncommon biochemical reactions in fungal secondary metabolism. Both enantiomers, marilines A1 **(159)** and A2 **(160)** inhibited human leukocyte elastase (HLE) with an IC_50_ value of 0.86 μM (Almeida et al., 2012).

## Compounds from other marine derived fungus

**Figure 6:**
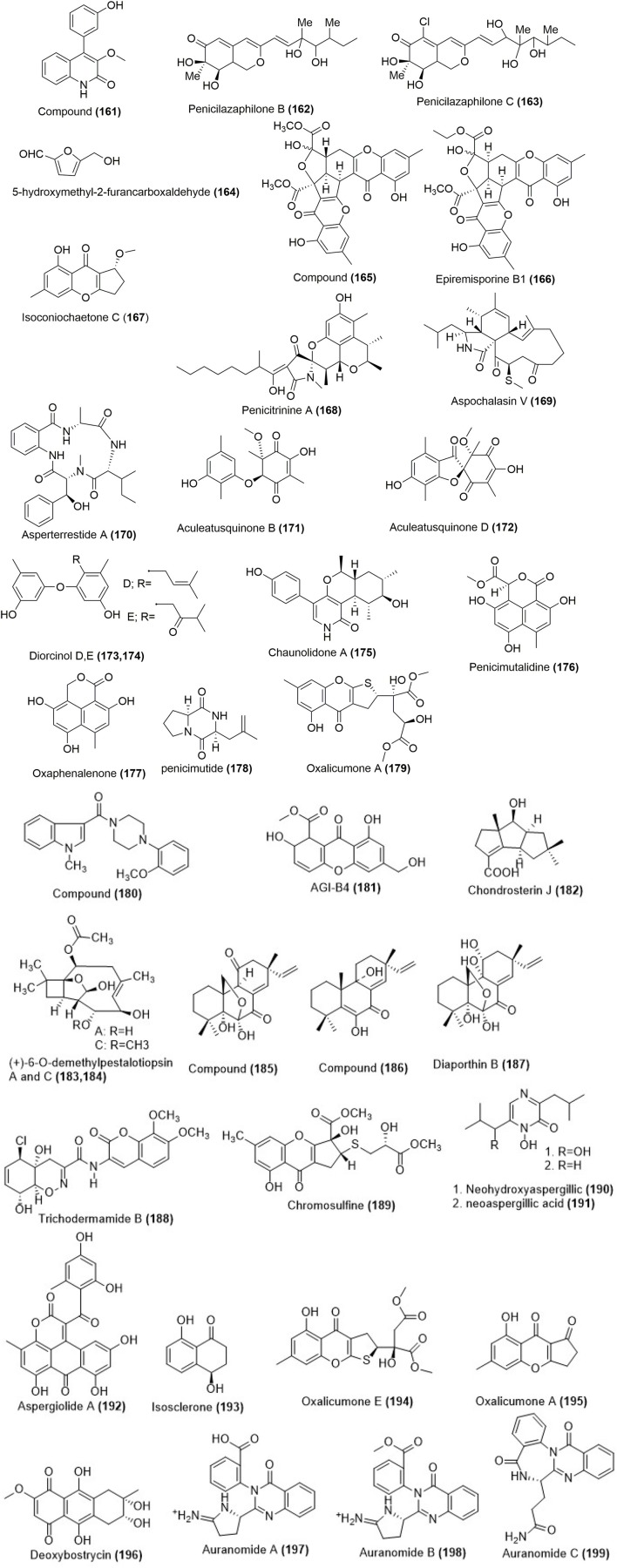
Chemical structures of metabolites isolated from other marine derived fungi. For complete chemical names, please see Supplementary Table 1.

*Aspergillus versicolor* Y31-2, which was obtained from seawater samples in the Indian Ocean, gave a quinolinone derivative **(161)** as shown in Figure 6. Compound **(161)** was cytotoxic against MCF-7 and SMMC-7721 cell lines with the IC_50_ values of 16.6 and 18.2 μmol/L (Li P. et al., 2016). Fermented products of marine fungus *Penicillium sclerotiorum* M-22 which was isolated from a rotten leaf sample collected on the west coast of Haikou, Hainan province, China gave two azaphilonidal derivatives penicilazaphilones B **(162)** and C **(163)** as shown in Figure 6. Cytotoxicity studies revealed that penicilazaphilones B **(162)** and C **(163)** were selective against melanoma cells B-16 and human gastric cancer cells SGC-7901 with the IC_50_ values of 0.29, 0.44 and 0.06, 0.72 μM respectively. The control experiments with normal mammary epithelial cells M10 at the same concentration did not show significant toxicity (Zhou et al., 2016). A furan derivative **(164)** was isolated from marine-derived fungus *Penicillium chrysogenum* HGQ6 which was obtained from Lianyungang sea mud sample (Figure 6). The compound **(164)** was active against BGC823 cell line with an IC_50_ value of 0.19 mg/mL, which was lower than that of adriamycin with an IC_50_ value of 0.06 mg/mL (Guo et al., 2016). A mutant from diethyl sulfate (DES) mutagenesis of a marine-derived fungus *Penicillium purpurogenum* G59 produced epiremisporine B **(165)**, epiremisporine B1 **(166)** and isoconiochaetone C **(167)** as shown in Figure 6. Epiremisporine B **(165)** exhibited cytotoxicity against K562, HL-60, with the IC_50_ values of 69.0 and 62.9 μg/mL. Similarly epiremisporine B1 **(166)** exhibited cytotoxicity against K562, HL-60 cell lines with the IC_50_ values of 53.1 and 54.7 μg/mL respectively while the percent inhibition rate for isoconiochaetone C **(167)** were 20.4 and 26.0 at 100 μg/ mL against K562 and HL-60 cell lines respectively (Xia et al., 2015). Penicitrinine A **(168)** a novel alkaloid with a unique spiro skeleton was isolated from a marine-derived fungus *Penicillium citrinum* (Figure 6). Penicitrinine A **(168)** showed toxicity against A-375, SPC-A1, and HGC-27 cancer cell lines with IC_50_ values of 20.1, 28.6 and 29.4 μM respectively. Morphological evaluation, apoptosis rate analysis, Western blot and real-time quantitative PCR (RT-qPCR) results showed that penicitrinine A could significantly induce A-375 cell apoptosis by decreasing the expression of Bcl-2 and increasing the expression of Bax. Additionally, anti-metastatic effects of penicitrinine A in A-375 cells by wound healing assay, trans-well assay, Western blot and RT-qPCR were also investigated. These results showed penicitrinine A significantly suppressed metastatic activity of A-375 cells by regulating the expression of MMP-9 and its specific inhibitor TIMP-1 (Liu Q. Y. et al., 2015).

*Aspergillus* sp. was found in the gut of a marine isopod *Ligia oceanica*, which was collected in the seaside of Dinghai in Zhoushan, Zhejiang Province of China, that produced aspochalasin V (**169**; Figure 6). Apochalasin V **(169)** showed moderate activity against PC3 and HCT116 cell line with the IC_50_ values of 30.4 and 39.2 μM respectively (Liu et al., 2014). Fungus *Aspergillus terreus* SCSGAF0162 was obtained from the tissue of gorgonian *Echinogorgia aurantiaca* collected in Sanya, Hainan Province, China which produced a cytotoxic and antiviral cyclic tetrapeptide asperterrestide A **(170)** as shown in Figure 6. Compound **(170)** was cytotoxic to human carcinoma U937 and MOLT4 cell lines with the IC_50_ values of 6.4 and 6.2 μM respectively (He et al., 2013). Aculeatusquinones B and D **(171, 172)** were produced from marine-derived fungus *Aspergillus aculeatus* (Figure 6). The chemical structures of these compounds were determined by spectroscopic methods. Compounds **(171)** and **(172)** were cytotoxic to HL-60, K562, and A-549 cell lines with the IC_50_ values in the range of 5.4–6.1 μM (Chen et al., 2013).

Diorcinol D **(173)** and diorcinol E **(174)** (Figure 6) were produced from the marine-derived fungus *Aspergillus versicolor*. Their chemical structures were determined using spectroscopic analysis. Compound **(173)** was moderately cytotoxic against HeLa and K562 cell lines with the IC_50_ values of 31.5 and 48.9 μM respectively while compound **(174)** showed cytotoxicity against only HeLa cell line with the IC_50_ value 36.5 μM (Gao et al., 2013b). A new pyridinone, chaunolidone A (**175**; Figure 6) was isolated from marine-derived fungus *Chaunopycnis* sp. (CMB-MF028) which was obtained from the inner tissue of a pulmonate false limpet *Siphonaria* sp. that was collected from rock surfaces in the intertidal zone of Moora Park, Shorncliffe, Queensland, Australia. Chaunolidone A **(175)** was found to be a selective and potent inhibitor of human non-small cell lung carcinoma cell NCI-H460 with the IC_50_ value 0.09 μM (Shang et al., 2015).

Penicimutalidine **(176)** and a known compound oxaphenalenone (**177**; Figure 6) were isolated from a fungal mutant generated through the diethyl sulfate (DES) mutagenesis of marine-derived *Penicillium purpurogenum* G59. The IC_50_ values for cytotoxicity of **(176)** and **(177)** on HL-60 cells under the same conditions were determined to be 95.2 μg/ mL (313.2 μM) and 14.0 μg/ mL (56.9 μM). Compounds **(176)** and **(177)** also weakly inhibited the K562 cells with inhibition rate (IR) % values of 20.8 and 28.1% at 100 μg/mL (328.9 μM for **176** and 406.5 μM for **177**). The positive control 5-fluorouracil inhibited K562 cells with an IR% of 40.3% at 100 μg/mL (796.2 μM) (Li C.-W. et al., 2016). A novel cyclic dipeptide, named penicimutide **(178)** was produced from a neomycin-resistant mutant of the marine-derived fungus *Penicillium purpurogenum* G59 (Figure 6). Penicimutide **(178)** was selective against the HeLa cells with an inhibition rate (IR%) of 39.4% at 100 μg/mL which was similar to that of the positive control 5-fluorouracil (IR% of 41.4% at 100 μg/mL against HeLa cells) (Wang et al., 2016).

Marine-derived fungus *Penicillium oxalicum* SCSGAF 0023, which was isolated from the South China Sea gorgonian *Muricella flexuosa*, produced oxalicumone A **(179**; (Figure 6). The compound **(179)** was cytotoxic against A375 and SW-620 cell lines with IC_50_ values of 11.7 and 22.6 μM (Sun et al., 2013). Compound (**180**; Figure 6) was isolated from the fungal strain *Aspergillus sydowii* SCSIO 00305 which was collected from a healthy tissue of *Verrucella umbraculum*. The compound **(180)** showed significant cytotoxicity against A375 cell lines with the IC_50_ value of 5.7 μM (He et al., 2012). A cytotoxic compound AGI-B4 (**181**; Figure 6) was obtained from the culture of a marine-derived fungus *Neosartorya fischeri* strain 1008F1. The chemical structure of the isolated compound was elucidated on the basis of spectroscopic data. Compound **(181)** showed toxicity aginst human gastric cancer cell line SGC-7901 with an IC_50_ value of 0.29 μM and against hepatic cancer cells BEL-7404 with an IC_50_ value of 0.31 μM (Tan et al., 2012). Fungus *Chondrostereum* sp. which was collected from soft coral *Sarcophyton tortuosum* in Hainan Sanya National Coral Reef Reserve, China produced chondrosterin J (**182**; Figure 6). The chemical structure of the compound was determined using NMR, mass spectrometry and single crystal X-ray diffraction techniques. The compound **(182)** was cytotoxic against human nasopharyngeal cancer cell lines CNE-1 and 2 with the inhibitory concentration (IC_50_) values of 1.32 and 0.56 μM respectively (Li H.-J. et al., 2014). Fungus *Ascotricha* sp. ZJ-M-5 was obtained from a mud sample in Fenghua, China which produced compound **(183)** and (+)-6-O-demethylpestalotiopsin C (**184**; Figure 6). Compounds **(183)** and **(184)** were cytotoxic against HL-60 and K562 with the IC_50_ values 6.9 and 12.3 μM respectively (Wang W.-J. et al., 2014).

Fungal strain HS-1 was isolated from the sea cucumber *Apostichopus japonicas* that gave two pimarane diterpenoids **(185, 186)** and a known compound diaporthin B **(187)** as shown in (Figure 6). Their chemical structures and absolute configurations were determined using NMR and CD experiments. Compounds **(185-187)** were effective in growth inhibition of KB and KBv200 cell lines with the IC_50_ values of 3.51, 2.34 μg/mL, 20.74, 14.47 μg/mL, and 3.86, 6.52 μg/mL respectively (Xia et al., 2012).

The trichodermamides are modified dipeptides isolated from a wide variety of fungi, including *Trichoderma virens*. Previous studies have reported that trichodermamide B initiated cytotoxicity in HCT-116 colorectal cancer cells. In the present study trichodermamide B (**188**; Figure 6) showed an IC_50_ value of 3.1 μM in HeLa cell line. Compound **(189)** caused S-phase accumulation and cell death in HeLa cells, suggesting response to DNA double strand breaks (Jans et al., 2017). Chromosulfine (**189**; Figure 6), a novel cyclopentachromone sulfide, was isolated from a neomycin-resistant mutant of the marine-derived fungus, *Penicillium purpurogenum* G59. Its structure, including stereochemistry, was determined using spectroscopic methods using NMR, electronic CD (ECD) analysis and Mosher's method. The compound **(189)** showed toxicity against K562, HL-60, BGC-823, HeLa, and MCF-7 cell lines with IC_50_ values of 60.8, 16.7, 73.8, 75.4, and 59.2 μM (Yi et al., 2016).

Neohydroxyaspergillic **(190)** and neoaspergillic acid **(191)** (Figure 6) were isolated from the marine-derived fungus (strain CF07002) of the genus *Aspergillus*. Their structures were determined by the interpretation of NMR spectroscopic data which were corroborated by subsequent synthesis. Compound **(191)** exhibited toxicity against Jurkat, K562, U937, and Raji cell lines with the IC_50_ values of 31.6, 50.1, 42.6, and 54.9 μM respectively. Compound **(190)** was poorly active against Jurkat cell lines with an IC_50_ value of 60.2 μM (Cardoso-Martinez et al., 2015). *Aspergillus glaucus* was obtained from the marine sediment in Fujian province of the People's Republic of China which gave a novel anthraquionone derivative aspergiolide A **(192)**. The active components of this fungus were isolated which resulted in the identification of a novel naphtho[1,2,3-de]chromene-2,7-dione skeleton. Compound **(192)** acts by topoisomerase II inhibition similar to adriamycin activity. Further experiments with BEL-7402 cells showed that **(192)** reduced cancer growth via a caspase dependent pathway (Wang Y. et al., 2014). Marine-derived fungus, *Aspergillus fumigatus* was isolated from marine green algae in Seosaeng-myeon, Ulsan in the Republic of Korea which produced isosclerone **(193)** as shown in Figure 6. It showed cytotoxicity toward MCF-7 human breast cancer cells with the IC_50_ value 63 μM after 24 h incubation. Further experiments showed that compound **(193)** inhibited the protein and gene expressions of MMP-2,-9 in MCF-7 human breast cancer cells by altering MAPK signaling pathway (Li Y.-X. et al., 2014). Marine gorgonian-associated fungus *Penicillium oxalicum* SCSGAF 0023 produced oxalicumone E **(194)** and oxalicumone A (**195**; Figure 6). The chemical structures of these compounds were determined by spectroscopic analysis. Compounds **(194)** and **(195)** exhibited cytotoxicity against eight cell lines (H1975, U937, K562, BGC823, MOLT-4, MCF-7, HL60, and Huh-7) with the IC_50_ values of <10 μM respectively (Bao et al., 2014). Deoxybostrycin **(196)** is an anthraquinone compound which was obtained from the marine mangrove fungus *Nigrospora* sp. No. 1403 as shown in Figure 6. The *in vitro* cytotoxicity of deoxybostrycin against MDA-MB-435, HepG2, and HCT-116 cancer cell lines were determined with the IC_50_ values of 3.1, 29.9, and 5.6 μM respectively (Chen et al., 2012). Three new alkaloids auranomides A and B **(197**, **198)** and auranomide C **(201)** were isolated from the marine-derived fungus *Penicillium aurantiogriseum* (Figure 6). The chemical structures of compounds **(197-199)** were elucidated by using spectroscopic methods such as IR, high-resolution mass spectroscopy and two-dimensional NMR spectroscopy. Auranomides A-C **(197-199)** exhibited moderate cytotoxic activity against K562, ACHN, HEPG2, and A549 cell lines. Auranomide B **(199)** displayed the best activity among them with an IC_50_ value of 0.097 μmol/mL against HEPG2 cells (Song et al., 2012).

## An overview of cytotoxicity results

As discussed before in different sections in this review article, a total of 199 compounds isolated from marine fungi have shown considerable promise as cytotoxic agents with potential to be developed as anticancer agents in recent years. About half of these compounds have been known to be isoloated from terrestrial or other natural sources previously but they have been reported to be isolated from the marine soruces for the first time. The Supplementary Table 1 outlines the novelty of these compounds with known previous anticancer acitivities, if any. A number of compounds reported in this review article have shown considerable anticancer activity comparable to positive controls (which are currently used anticancer drugs). Many of these metabolites have displayed inhibitory concentrations in the low micromolar range which obviously mark their potential to be developed as anticancer drugs. However, there is a definite need to improve these inhibitory concentrations since lower dosage would help in eliminating undesired side effects. The exploration in this direction has to be a two pronged approach: one, where the actual cellular targets that lead to cytotoxic effects need to be identified while the other, needs to focus on identifying the structural moieties that are responsible for cytotoxicity. The latter effort would lead to structure-activity based drug design programs to alter chemical functionalities in order to achieve higher efficacy.

Additionally, since several of these metabolites possess structural features (such as compounds **84**, **88**, **116**, **119**, **129**, **131**, **192**, and **196**) that would enable binding to DNA and RNA, cancer targets that involve nucleic acid recognition should be probed. For example, chromosomal DNA ends in humans, which are rich in guanines, have been shown to form a unique four stranded structures called G-quadruplexes. Both *in vitro* and *in vivo* studies have shown the formation of these non-canonical structures which use assembly of four guanines (called G-tetrads), hydrogen bonded in a Hoogsteen fashion (Ranjan et al., 2010). After every cell division, the telomeric DNA gets shortened by certain bases and this process continues until reaching a threshold (called Hayflick limit) where senescence is initiated in a normal cell cycle. However, in the majority of cancer cases, a ribonucleoprotein called telomerase gets activated. The telomerase contains an RNA unit complementary to human telomeric repeat sequence TTAGGG. Binding of this RNA unit of telomerase initiates reverse transcription process to regenerate the curtailed telomere (Camarena et al., 2007; Fakhoury et al., 2007). Many research efforts have, therefore, targeted inhibiting/disrupting telomerase interaction with the telomeric DNA as a means to develop new therapies for cancer treatment (Mergny and Hélène, 1998). One of the ways in which this inhibition can be achieved, is by folding the telomeric ends as stable G-quadruplex structures since telomerase recognizes only the linear form of the telomeric DNA. As a result, small molecules that target these G-quadruplex structures have been tested to see if they could function as inhibitors of telomerase interaction. A number of small molecule inhibitors have been reported that bind to G-quadruplexes and enhance their stability (Xue et al., 2011; Ranjan and Arya, 2013; Ranjan et al., 2013). Such stabilizations are known to disfavor telomerase binding and thereby stopping the telomere regeneration. An important feature of G-quadruplex stabilization by small molecule is making stabilizing interactions with the G-tetrads by means of π-bonding. Some other molecules have shown interactions exclusively with the grooves whereas few have shown interactions both with the tetrads and the grooves. Several of the molecules reported in this review have features that would enable binding both with the G-tetrads and the grooves (for example compound **116**). Furthermore, topoisomerases are enzymes that remove supercoiling in DNA during the replication process and repairs strand breaks (Tse-Dinh, 2009; Pommier, 2013). Human DNA topoisomerase has been an attractive cancer target and anticancer drug camptothecin is known to elicit its effect by forming a ternary complex between the enzyme and the DNA. Some of the molecules reported in this review (e.g., compound **194**) have already shown topoisomerase inhibitory function. Stalling topoisomerase functions by its stabilization with small molecules is another target for anticancer therapy. Since several of these molecules possess structural features that would enable binding to nucleic acids, a screen that targets all forms of nucleic acid structures should be done. This would not only identify the lead compounds for cancer therapy but would also result in identifying the compound that could be of potential use in antibacterial and antiviral therapy.

In addition to this, there are many new discoveries that could have protein targets within the cancer cells. In fact, the majority of FDA approved anticancer drugs target proteins such as cyclin dependent kinases and histone deacytylase. Could these proteins targets be the potential mechanism by which these metabolites induce cytotoxicity? For some of the compounds (**64**, **65**, **168**), Bcl-2 downregulation was established as one factor that led to the apoptosis. Do these metabolites function by upregulation of tumor suppressor proteins such as p53 and Bax? The curiosities can be answered only when all protein targets are screened for binding; at least for the ones whose cellular functioning is fairly understood. This would usher a new beginning in the development of natural product based small molecules and possibly identify structural motifs that target specific regions in the protein binding pockets. Such leads can then be used to launch structure activity relationship programs to improve the potency of these leads whose inhibitory concentrations are mostly in the micromolar range. Overall, the discoveries presented in this review highlights many structural classes including some new skeletons that these metabolites produce, which have potential to be developed as clinically useful anticancer drugs. However, in the absence of more detailed studies that focuses on deciphering the cellular events that lead to cell death, their true potential as an anticancer compound might remain to be under-appreciated.

## Conclusions and perspectives

Marine life has been the source of several clinically useful drugs. The findings covered in this review highlight the discoveries of many new natural small molecules, some of them with novel skeletons, that have anticancer activity against a variety of cancer cell lines. The anticancer activity of these compounds is varied with inhibitory concentrations ranging from low to high micromolar concentrations. Some of these metabolites have inhibitory concentrations comparable or better than some of the currently used anticancer drugs. Clearly, these leads have not been explored in detail to determine the actual cellular targets that result in the cytotoxicity and that has been an area whose complete exploration may result in a paradigm shift in the current drug discovery efforts. However, other parallel efforts are needed to facilitate and accentuate marine based drug discovery. One such need is setting-up national and international centers for culture collection since many of the new metabolites reported here have been collected from harsh and hostile environments where the human reach is not easily achievable. This would also help not just in retaining these precious cultures but also in allowing wider reach of these metabolites to specialized groups. A major impediment in marine based drug discovery and, in general, natural product based drug discovery has been the lack of centers that foster programs at the interface of chemistry and biology. Clearly, such specialized centers that have expertise both in chemistry and biology could help in realizing true properties of these metabolites. Morever, lack of complete taxonomy details of the new species and bureaucratic difficulties in the implementation of Nagoya protocol hinder smooth access of knowledge and resources. Therefore, international agreements that clearly address these problems and seek solutions to it, could also greatly help in the smooth exchange of resources. Another important improvement in the area would be developing sustainable biochemical production processes of the screening hits as demonstrated in the case of anticancer compounds Scopularide A and B (Yu et al., 2008; Kramer et al., 2014). Additionally, efforts should also be initiated to look beyond anticancer properties of these molecules.

## Author contributions

SD, VP, and NR reviewed the contents critically. VP and NR drew chemical structures and assisted in the preparation of Supplementary Table 1. SD and NR wrote the review.

### Conflict of interest statement

The authors declare that the research was conducted in the absence of any commercial or financial relationships that could be construed as a potential conflict of interest.
