# Supplementary material for: Marine Fungi: A Source of Potential Anticancer Compounds
Source: Front Microbiol. 2018 Jan 5;8:2536. doi: 10.3389/fmicb.2017.02536 (PMC5760561; doi:10.3389/fmicb.2017.02536)
Supplement: Supplementary file 1 [file Table1.pdf]

Supplementary Table 1. Novel anticancer bioactive compounds reported from marine fungi

**Sr. No.**	**Crude extract/isolated metabolite**	**New / Known**	**Known Source/ Activity**	**Endophytic fungal strain**	**Host plant(s) (family),**	**Plant part or tissue Locality of host plants**	**Cell line**	**(IC50)/Inhibition**	**References**
1	Simplicilliumtides A **(1)** and G **(3)**	New		*Simplicillium obclavatum* EIODSF 020	Deep Sea Sediments	East Indian Ocean	HL-60, K562	64.7 and 100 μM	Liang et al. 2016
2	Simplicilliumtides E **(2)** and H **(4)**	New		*Simplicillium obclavatum* EIODSF 020	Deep Sea Sediments	East Indian Ocean	K562	39.4 and 73.5 μM	Liang et al. 2016
3	Acaromycin A **(5)**	New		*Acaromyces ingoldii* FS121	Deep Sea Sediments	South China Sea	MCF-7, NCI-H460, SF-268, and HepG-2	<10 μM	Gao et al. 2016
	(+)-cryptosporin **(6)**	Known	Marine sediments *Streptomyces* sp. SCSIO 03219	*Acaromyces ingoldii* FS121	Deep Sea Sediments	South China Sea	MCF-7, NCI-H460, SF-268, and HepG-2	<10 μM	Gao et al. 2016
4	Asperolide E **(7)**	New		*Aspergillus wentii* SD-310	Deep Sea Sediments	South China Sea	HeLa, MCF-7, and NCI-H446	10.0, 11.0, and 16.0 μM	Li et al. 2016a
5	Asperethers A-E **(8-12)**	New		*Aspergillus wentii* SD-310	Deep Sea Sediments	South China Sea	A549	20, 16, 19, 17, and 20 μM	Li et al. 2016b
6	Circumdatin G **(13)**	Known	Marine Derived fungi No cytotoxicity	*Aspergillus westerdijkiae* SCSIO 05233	Deep Sea Sediments	South China Sea	K562, HL-6	25.8–44.9 μM	Fredimoses et al. 2015

7	Xanthocillin X **(14)** ,	known /	*Aspergillus* sp. Mycotoxin / Marine cytotoxic	*Penicillium commune* SD-44	Deep Sea Sediments	South China Sea	MCF-7, HepG2, NCI-H460, HeLa, DU145, and MDA-MB-231,	12.0, 7.0, 10.0, 10.0, 8.0, and 8.0 µg/mL	Shang et al. 2012;
7	Chrysogine **(15)**	known	Terrestrial mycotoxin / Marine cytotoxic	*Penicillium commune* SD-44	Deep Sea Sediments	South China Sea	SW1990,	20.0 µg/mL	Zhao et al. 2012
7	Meleagrin **(16)**	known	Terrestrial mycotoxin / Marine cytotoxic	*Penicillium commune* SD-44	Deep Sea Sediments	South China Sea	DU145,HepG2, NCIH460, HeLa, and MDA-MB-231	5.0,12.0, 22.0, 20.0, and 11.0 µg/mL	Zhao et al. 2012
8	Compound **(17)**	New		*Penicillium* sp. PR19 N-1	Deep Sea Sediments	Antarctic deep-sea	HL-60, A-549	28.3–45.8 µM	Lin et al. 2014
8	Eremofortine C **(18)**	New		*Penicillium* sp. PR19 N-1	Deep Sea Sediments	Antarctic deep-sea	HL-60, A-549	5.2–82.8 µM	
9	Aspiketolactonol (**19**), Aspilactonols A–F (**20-25**), Aspyronol (**26**), and Epiaspinonediol (**27**)	New		*Aspergillus* sp. 16-02-1	Deep-sea sediment	Pacific Ocean	K562, HL-60, HeLa, and BGC-823	-	Chen et al. 2014b
	(S)-2-(2′-hydroxyethyl)-4-methyl-γ-butyrolactone **(28),**	Known	Plant *Annona muricata* not cytotoxic / marine cytotoxic	*Aspergillus* sp. 16-02-1	Deep-sea sediment	Pacific Ocean	K562, HL-60, HeLa, and BGC-823	-	Chen et al. 2014b
	Dihydroaspyrone (**29**),	Known	Marine not cytotoxic	*Aspergillus* sp. 16-02-1	Deep-sea sediment	Pacific Ocean	K562, HL-60, HeLa, and BGC-823	-	Chen et al. 2014b

	Aspinotriol A (**30**),	Known	Marine not cytotoxic	*Aspergillus* sp. 16-02-1	Deep-sea sediment	Pacific Ocean	K562, HL-60, HeLa, and BGC-823	-	Chen et al. 2014b
	Aspinotriol B (**31**),	Known	Marine not cytotoxic	*Aspergillus* sp. 16-02-1	Deep-sea sediment	Pacific Ocean	K562, HL-60, HeLa, and BGC-823	-	Chen et al. 2014b
	Chaetoquadrin F **(32)**	Known	Terrestrial / Marine cytotoxic	*Aspergillus* sp. 16-02-1	Deep-sea sediment	Pacific Ocean	K562, HL-60, HeLa, and BGC-823	-	Chen et al. 2014b
	cyclo(D-Pro-D-Phe) **(33)**,	Known	Marine bactria no cytotoxic	*Aspergillus versicolor* ZBY-3	Deep-sea sediment	South east Pacific sea	K562	IR% 54.6 at 100 µg/mL	Dong et al. 2014
	cyclo(D-Tyr-D-Pro) **(34)**,	Known	Isolated from a natural source no cytotoxic	*Aspergillus versicolor* ZBY-3	Deep-sea sediment	South east Pacific sea	K562	IR% 72.9 at 100 µg/mL	Dong et al. 2014
10	phenethyl 5-oxo-L-prolinate **(35)**,	New		*Aspergillus versicolor* ZBY-3	Deep-sea sediment	South east Pacific sea	K562	IR% 23.5 at 100 µg/mL	Dong et al. 2014
	cyclo(L-Ile-L-Pro) **(36)**,	Known	From bacteria not cytotoxic	*Aspergillus versicolor* ZBY-3	Deep-sea sediment	South east Pacific sea	K562	IR% 29.6 at 100 µg/mL	Dong et al. 2014
	cyclo(L-Leu-L-Pro) **(37)**,	Known	From endophytic actinomycetes (cytotoxic), *Phellinus igniarius* (cytotoxic)	*Aspergillus versicolor* ZBY-3	Deep-sea sediment	South east Pacific sea	K562	IR% 30.9 at 100 µg/mL	Dong et al. 2014
	3β,5α,9α-trihydroxy-(22E,24R)-ergosta-7,22-dien-6-one **(38)**	known	Mushroom *Armillaria lueo-virens,* Not cytotoxic	*Aspergillus versicolor* ZBY-3	Deep-sea sediment	South east Pacific sea	K562	IR% 51.1 at 100 µg/mL	Dong et al. 2014

11	Engyodontiumones H **(39)**	New	Soil sample , *Aspergillus* sp. Y80118	*Engyodontium album* DFFSCS021	Deep-sea sediment	South China Sea	U937	4.9 μM	Yao et al. 2014
	Polyketides- AGI-B4 **(40)**	known	Soil sample , *Aspergillus* sp. Y80118	*Engyodontium album* DFFSCS021	Deep-sea sediment	South China Sea	U937	8.8 μM	Yao et al. 2014
11	Compound (**41**)	New		*Penicillium sp.* PR19N-1	Antarctic deep-sea sediment	Antarctic	HL-60 and A549	11.8 ± 0.2, 12.2 ± 0.1 μM	Wu et al. 2013
12	Wentilactone A **(42)**	Known	*Aspergillus wentii* Wehmer (NRRL 6435) from peanuts	*Aspergillus dimorphicus* SD317	Deep-sea sediment	South China Sea	Induce apoptosis and G2/M arrest of human lung carcinoma cells		Xu et al. 2015
	Wentilactone B **(43)**	Known	*Aspergillus wentii* Wehmer (NRRL 6435) from peanuts	*Aspergillus dimorphicus* SD317	Deep-sea sediment	South China Sea	Induce apoptosis and G2/M arrest of human lung carcinoma cells		Xu et al. 2015
13	Compound (**44**), Compound (**45**), Compound (**46**),	known	Terrestrial than marine	*Paecilomyces lilacinus* ZBY-1	Deep Sea	South China Sea	K562, MCF-7, HL-60, and BGC-823	22.3-139.0 μM	Cui et al. 2013
	Cerebroside A-D (**47-50**)	known	Terrestrial than marine	*Paecilomyces lilacinus* ZBY-1	Deep Sea	South China Sea	K562, MCF-7, HL-60, and BGC-823	22.3-139.0 μM	Cui et al. 2013
14	5-Chlorosclerotiamide **(51),**	New		*Aspergillus westerdijkiae* DFFSCS013	Deep-sea sedimment	South China Sea	K562	44 μM	Peng et al. 2013
	10-epi-sclerotiamide **(52)**	New		*Aspergillus westerdijkiae* DFFSCS014	Deep-sea sedimment	South China Sea	K563	53 μM	Peng et al. 2013

15	Luteoalbusin A **(53),**	New		*Acrostalagmus luteoalbus* SCSIO F457	Deep-sea sediment	South China Sea	SF-268 MCF-7 NCI-H460 HepG-2	0.46 , 0.23 , 1.15 , 0.91 µM	Wang et al. 2012
	Luteoalbusin B (**54)**	New		*Acrostalagmus luteoalbus* SCSIO F458	Deep-sea sediment	South China Sea	SF-268 MCF-7 NCI-H460 HepG-2	0.59 , 0.25 , 1.29 µM	Wang et al. 2012
	T988A **(55)**	known	Terrestrial Cytotoxic /Marine cytotoxic	*Acrostalagmus luteoalbus* SCSIO F459	Deep-sea sediment	South China Sea	SF-268 MCF-7 NCI-H460 HepG-2	Range of 0.91 to 17.78 µM	Wang et al. 2012
	Gliocladine C **(56)**	known	Terrestrial / Marine cytotoxic	*Acrostalagmus luteoalbus* SCSIO F460	Deep-sea sediment	South China Sea	SF-268 MCF-7 NCI-H460 HepG-2	Range of 0.91 to 17.78 µM	Wang et al. 2012
	Gliocladine D **(57)**	known	Terrestrial / Marine cytotoxic	*Acrostalagmus luteoalbus* SCSIO F461	Deep-sea sediment	South China Sea	SF-268 MCF-7 NCI-H460 HepG-2	Range of 0.91 to 17.78 µM	Wang et al. 2012
16	Brevione I-**(58)**,	New		*Penicillium* sp	Deep Sea Sedimments	East Pacific Ocean	MCF-7	7.44	Li et al. 2012
	Brevione A **(59)**	known	Terrestrial No cytotoxic / Marine cytotoxic	*Penicillium* sp	Deep Sea Sedimments	East Pacific Ocean	MCF-7	28.4 µM	Li et al. 2012
	Brevione I-**(58)**,	New		*Penicillium* sp	Deep Sea Sedimments	East Pacific Ocean	A549	32.5 µM	Li et al. 2012
17	Varioloid A **(60),**	New		*Paecilomyces variotii* EN-291	*Grateloupia turuturu*, a marine red alga	Coast of Qingdao, P. R. China,	A549, HCT116, HepG2	Range of 2.6 - 8.2 µg/mL	Zhang et al. 2016
	Varioloid B **(61)**	New		*Paecilomyces variotii* EN-291	*Grateloupia turuturu*, a marine red alga	Coast of Qingdao, P. R. China,	A549, HCT116, HepG2	Range of 2.6 - 8.2 µg/mL	Zhang et al. 2016

18	6β,9α-dihydroxy-14-p-nitrobenzoylcinnamolide **(62)**,	New		*Aspergillus ochraceus* Jcma1F17	Marine alga *Coelarthrum* sp.	Paracel Islands, South China Sea	H1975, U937, K562, BGC-823, Molt-4, MCF-7, A549, HeLa, HL60, and Huh-7	Range of 1.95 to 6.35 μM	Fang et al. 2014
18	Insulicolide A **(63)**	known	Terrestrial / Marine cytotoxic	*Aspergillus ochraceus* Jcma1F17	Marine alga *Coelarthrum* sp.	Paracel Islands, South China Sea	H1975, U937, K562, BGC-823, Molt-4, MCF-7, A549, HeLa, HL60, and Huh-7	Range of 1.95 to 6.35 μM	Fang et al. 2014
19	Physcion **(64)**	known	Both terrestrial and marine sources Cytotoxic	*Microsporum* sp.	Marine red alga *Lomentaria catenata*,	Guryongpo, NamGu, PoHang in Republic of Korea	HeLa	Induces apoptotic cell death in HeLa cells in the association with activating expressions of caspase family enzymes and p53 followed by Bax and Bcl-2 regulation.	Wijesekara et al. 2014

20	Neoechinulin A **(65)**	Known	/Marine derived fungi	*Microsporum* sp.	Marine red alga Lomentaria catenata,	Guryongpo, NamGu, PoHang in Republic of Korea	HeLa Cell lines	Induce cell apoptosis through down-regulating of Bcl-2 expression, up-regulating of Bax expression, and activating the caspase-3 pathway	Wijesekara et al. 2013
21	Asperolide A B **(66, 67)**	New		*Aspergillus wentii* EN-48	*Sargassum* sp	China Sea	HeLa, HepG2, MCF-7, MDA-MB-231, NCI-H460, SMMC-7721, and SW1990	Moderate activity. In the range of 28 - 97 µM	Sun et al. 2012
	Tetranorditerpenoid derivative **(68)**	known	Terrestrial Fungus *Sclerotinia homoeocarpa*,no cytotoxic	*Aspergillus wentii* EN-48	*Sargassum* sp	China Sea	HeLa, HepG2, MCF-7, MDA-MB-231, NCI-H460, SMMC-7721, and SW1990	Moderate activityIn the range of 28 - 97 µM	Sun et al. 2012
	Wentilactone A **(42)**	Known,	*Aspergillus wentii* Wehmer (NRRL 6435) from peanuts	*Aspergillus wentii* EN-48	*Sargassum* sp	China Sea	HeLa, HepG2, MCF-7, MDA-MB-231, NCI-H460, SMMC-7721, and SW1990	Moderate activityIn the range of 28 - 97 µM	Sun et al. 2012

	Wentilactone B **(43)**	Known	*Aspergillus wentii* Wehmer (NRRL 6435) from peanuts	*Aspergillus wentii* EN-48	*Sargassum* sp	China Sea	HeLa, HepG2, MCF-7, MDA-MB-231, NCI-H460, SMMC-7721, and SW1990	Moderate activity In the range of 28 - 97 µM	Sun et al. 2012
	Wentilactoneand B **(43)**	Known	*Aspergillus wentii* Wehmer (NRRL 6435) from peanuts	*Aspergillus wentii* EN-48	*Sargassum* sp	China Sea	HeLa, HepG2, MCF-7, MDA-MB-231, NCI-H460, SMMC-7721, and SW1990	17 µM	Sun et al. 2012
22	Spirobrocazines C **(69)**	New		*Penicillium brocae* MA-231	Mangrove plant *Avicennia marina*	Hainan Island, P. R. China	A2780	59 µM	Meng et al. 2016
	Brocazine G **(70)**	New		*Penicillium brocae* MA-231	Mangrove plant *Avicennia marina*	Hainan Island, P. R. China	A2780 and A2780 CisR	664 nM , 661 nM	Meng et al. 2016
23	2,4-Dihydroxy-6-nonylbenzoate **(71)**	known	Merine cytotoxic	*Lasiodiplodia* sp. 318	*Excoecaria agallocha*	Guangdong Province, China	MMQ, GH3	5.29 µM, 13.05 µM	Huang et al. 2017
24	Chloropreussomerins A and B **(72,73)**	New		*Lasiodiplodia theobromae* ZJ-HQ1	*Acanthus ilicifolius*	Guangdong Province, China	A549 and MCF-7	Range of 5.9-8.9 µM	Chen et al. 2016
	preussomerin K **(74)**,	known	Endophytic fungus no cytotoxic	*Lasiodiplodia theobromae* ZJ-HQ1	*Acanthus ilicifolius*	Guangdong Province, China	A549, HepG2, MCF-7	Range of 2.5-9.4 µM	Chen et al. 2016

	Preussomerin H **(75)**	known	Endophytes no cytotoxic	*Lasiodiplodia theobromae* ZJ-HQ1	*Acanthus ilicifolius*	Guangdon g Province, China	A549, HepG2, MCF-7	Range of 2.5-9.4 μM	Chen et al. 2016
	Preussomerin G **(76)**	known	Corprophilus fungus *Preussia isomera* and the endophytic fungus *Harmonema dematioides* cytotoxic	*Lasiodiplodia theobromae* ZJ-HQ1	*Acanthus ilicifolius*	Guangdon g Province, China	A549, HepG2, MCF-7	Range of 2.5-9.4 μM	Chen et al. 2016
	Preussomerin F **(77)**	known	Coporophilous fungus no cytotoxic	*Lasiodiplodia theobromae* ZJ-HQ1	*Acanthus ilicifolius*	Guangdon g Province, China	A549, HepG2, MCF-7	Range of 2.5-9.4 μM	Chen et al. 2016
25	7-O-methylnigrosporolide **(78)** Pestalotioprolide D-F **(79-81)**	New		*Pestalotiopsis microspore*	*Drepanocarpus lunatus*	Cameroon	L5178Y, A2780	0.7, 5.6, 3.4, and 3.9 μM	Liu et al 2016
	Pestalotioprolide E **(80)**	New		*Pestalotiopsis microspore*	*Drepanocarpus lunatus*	Cameroon	A2780	1.2 μM	Liu et al 2016
26	Campyridone D **(82)**	New		*Campylocarpon* sp. HDN13-307	*Sonneratia caseolaris*	PR China	HeLa	8.8 μM	Zhu et al. 2016
	Ilicicolin H **(83)**	Known	Terrestrial / Marine cytotoxic	*Campylocarpon* sp. HDN13-307	*Sonneratia caseolaris*	PR China	HeLa	4.7 μM	Zhu et al. 2016
27	Dihydroaltersolanol C **(84)**,	New		*Stemphylium globuliferum*	*Avicennia marina*	Hurghada in Egypt	L5178Y	3.4 μM	Moussa et al. 2016, Liu et al. 2015, Debbab, et al. 2012
	Altersolanol A **(85)**,	known	Terrestrial, Cytotoxic Endophytes	*Stemphylium globuliferum*	*Avicennia marina*	Hurghada in Egypt	L5178Y	2.53 μM	Moussa et al. 2016, Liu et al. 2015,

									Debbab, et al. 2012
	Altersolanol B **(86)**	known	Terrestrial, Cytotoxic Endophytes	*Stemphylium globuliferum*	*Avicennia marina*	Hurghada in Egypt	L5178Y	3.78 µM	Moussa et al. 2016, Liu et al. 2015, Debbab, et al. 2012
	Alterporriol E **(88),**	New		*Stemphylium globuliferum*	*Avicennia marina*	Hurghada in Egypt	L5178Y	6.9 µM	Moussa et al. 2016, Liu et al. 2015, Debbab, et al. 2012
	Altersolanol N **(87)**	known	Terrestrial, Cytotoxic Endophytes	*Stemphylium globuliferum*	*Avicennia marina*	Hurghada in Egypt	L5178Y	Low micromolar range (% growth - 1.4)	Moussa et al. 2016, Liu et al. 2015, Debbab, et al. 2012
28	Rhizovarins A, B, E (89, 90, 91),	New		*Mucor irregularis* QEN-189	*Rhizophora stylosa*	Hainan Island, China	A-549	11.5, 6.3, and 9.2 , µM	Gao et al. 2016
	Penitrems A,C F **(92,93,94)**,	known	Terrestrial mycotoxin / Marine cytotoxic	*Mucor irregularis* QEN-189	*Rhizophora stylosa*	Hainan Island, China	A-549	8.4, 8.0, and 8.2 µM	Gao et al. 2016
	3β-hydroxy- 4β-desoxypaxilline **(95)**	known	Marine cytotoxic	*Mucor irregularis* QEN-189	*Rhizophora stylosa*	Hainan Island, China	A-549	4.6 µM	Gao et al. 2016
	Rhizovarin A B **(89,90)**,	New		*Mucor irregularis* QEN-189	*Rhizophora stylosa*	Hainan Island, China	HL-60	9.6, 5.0, µM	Gao et al. 2016
	Penitrems A,C F **(92,93,94)**,	known	Terrestrial mycotoxin / Marine cytotoxic	*Mucor irregularis* QEN-189	*Rhizophora stylosa*	Hainan Island, China	HL-60	7.0,4.7, 3.3 µM	Gao et al. 2016

	3β-hydroxy- 4β-desoxypaxilline **(95)**	known	Known / Marine cytotoxic	*Mucor irregularis* QEN-189	*Rhizophora stylosa*	Hainan Island, China	HL-61	2.6 μM	Gao et al. 2016
29	Pestalpolyol I **(96)**	New		*Pestalotiopsis clavispora*	*Rhizophora harrisonii*	Port Harcourt (Nigeria)	L5178Y	4.10 μM	Perez et al. 2016
30	Rhytidchromones A, B,C and E, **(97,98,99,100)**	New		*Rhytidhysteron rufulum*	*Bruguiera gymnorrhiza*	Prachuab Kiri Khan Province, Thailand	Kato-3	16.0 to 23.3 μM	Chokpaiboon et al. 2016
	Rhytidchromones A, C **(97, 99)**	New		*Rhytidhysteron rufulum*	*Bruguiera gymnorrhiza*	Prachuab Kiri Khan Province, Thailand	MCF-7	19.3 and 17.7 μM	Chokpaiboon et al. 2016
31	Ethyl-2,4-dihydroxy-6-(80 -hydroxynonyl)-benzoate **(101)**	New		*Lasiodiplodia* sp. 318	*Excoecaria agallocha*	Guangdong Province, China	MDA-MB-435, HepG2,HCT-116, A549, THP1	10.13, 12.50, 11.92, 13.31 , 39.74 μM	Li et al. 2016c
32	Beauvericin **(102)**	Known	Terrestial No cytotoxic , Marine cytotoxic	*Fusarium* sp. (No. DZ27)	Mangrove-derived endophytic fungus	South China Sea	KB and KBv200	5.76 ± 0.55, 5.34 ± 0.09 μM	Tao et al. 2015
33	4-(2',3'-dihydroxy-3'-methyl-butanoxy)-phenethanol **(103)**	New		*Penicillium* sp.FJ-1	*Avicennia marina*	Fujian, China	Tca8113 and MG-63	26 , 35 μM	Zheng et al. 2014
33	15- hydroxy-6α,12-epoxy-7β,10αH,11βH-spiroax-4-ene-12-one **(104)**	New		*Penicillium* sp.FJ-2	*Avicennia marina*	Fujian, China	Tca8113, WRL-68, MG-63	10, 58 μM, 55 nM	Zheng et al. 2014
34	3,4-seco-sonderianol **(105)**	Known	*Trigonostemon chinensis* plant antimicrobial Marine cytotoxic	Endophytic fungus J3	Mangrove plant *Ceriops tagal*	Hainan province, China	K562, SGC-7901, BEL-7402	9.2 , 15.7 , 25.4 μg/mL	Zeng et al. 2015

35	Waol A **(106)**,	known	Terrestial No cytotoxic , Marine cytotoxic	*Acremonium strictum*	*Rhizophora apiculata*	Island of CatBa, Vietnam	human cisplatin-sensitive, A2780	27.1 µM	Hammerschmidt et al. 2014
	Pestalotiopene A **(107)**	known	Mangroove fungi, week cytotoxic , Marine cytotoxic	*Acremonium strictum*	*Rhizophora apiculata*	Island of CatBa, Vietnam	human cisplatin-sensitive, A2780	76.2 µM	Hammerschmidt et al. 2014
	Cytosporone E **(108)**	known	Endophytic fungi Cytospors, Marine cytotoxic	*Acremonium strictum*	*Rhizophora apiculata*	Island of CatBa, Vietnam	human cisplatin-sensitive, A2780	8.3 µM	Hammerschmidt et al. 2014
	Waol A **(106)**,	known	Terrestrial, Marine cytotoxic	*Acremonium strictum*	*Rhizophora apiculata*	Island of CatBa, Vietnam	human cisplatin-Resistant Resistant	12.6 µM	Hammerschmidt et al. 2014
	Pestalotiopene A **(107)**	known	Mangroove fungi, week cytotoxic , Marine cytotoxic	*Acremonium strictum*	*Rhizophora apiculata*	Island of CatBa, Vietnam	human cisplatin-Resistant Resistant	30.1 µM	Hammerschmidt et al. 2014
	Cytosporone E (108)	known	Endophytic fungi Cytospors, Marine cytotoxic	*Acremonium strictum*	*Rhizophora apiculata*	Island of CatBa, Vietnam	human cisplatin-Resistant Resistant	19.0 µM	Hammerschmidt et al. 2014
36	Dothiorelone F **(109),**	New		*Dothiorella* sp.	*Aegiceras corniculatum*	Fujian Province of China	Raji cancer	2 µg/mL	Du et al. 2014
	Dothiorelone G **(110)**	Known	(Cytospore B) Endophyte plant not identified No cytotoxic	*Dothiorella* sp.	*Aegiceras corniculatum*	Fujian Province of China	Raji cancer	2 µg/mL	Du et al. 2014

37	3β,5α-dihydroxy-(22*E*,24*R*)-ergosta-7,22-dien-6-one **(111)**,	Known	Fist time ntural source	*Aspergillus terreus* (No. GX7-3B)	*Bruguiera gymnoihiza* (Linn.)	South China Sea	MCF-7,	4.98 μM	Deng et al. 2013a
	3β,5α-dihydroxy-(22*E*,24*R*)-ergosta-7,22-dien-6-one **(111)**,	Known	Fist time ntural source	*Aspergillus terreus* (No. GX7-3B)	*Bruguiera gymnoihiza* (Linn.)	South China Sea	A549,	1.95 μM	Deng et al. 2013a
	3β,5α-dihydroxy-(22*E*,24*R*)-ergosta-7,22-dien-6-one **(111)**,	Known	Fist time ntural source	*Aspergillus terreus* (No. GX7-3B)	*Bruguiera gymnoihiza* (Linn.)	South China Sea	Hela and	0.68 μM	Deng et al. 2013a
	3β,5α-dihydroxy-(22*E*,24*R*)-ergosta-7,22-dien-6-one **(111)**,	Known	Fist time ntural source	*Aspergillus terreus* (No. GX7-3B)	*Bruguiera gymnoihiza* (Linn.)	South China Sea	KB	1.50 μM	Deng et al. 2013a
	Beauvericin **(102)**	Known	Terrestial cytotoxic , Marine cytotoxic	*Aspergillus terreus* (No. GX7-3B)	*Bruguiera gymnoihiza* (Linn.)	South China Sea	MCF-7,	2.02 μM	Deng et al. 2013a
	Beauvericin **(102)**	Known	Terrestial cytotoxic , Marine cytotoxic	*Aspergillus terreus* (No. GX7-3B)	*Bruguiera gymnoihiza* (Linn.)	South China Sea	A549,	0.82 μM	Deng et al. 2013a
	Beauvericin **(102)**	Known	Terrestial cytotoxic , Marine cytotoxic	*Aspergillus terreus* (No. GX7-3B)	*Bruguiera gymnoihiza* (Linn.)	South China Sea	Hela and	1.14 μM	Deng et al. 2013a
	Beauvericin **(102)**	Known	Terrestial cytotoxic , Marine cytotoxic	*Aspergillus terreus* (No. GX7-3B)	*Bruguiera gymnoihiza* (Linn.)	South China Sea	KB	1.10 μM	Deng et al. 2013a
	3β,5α,14α-trihydroxy-(22*E*,24*R*)-ergosta-7, 22-dien-6-one **(112)**,	Known	Terrestial No cytotoxic , Marine cytotoxic	*Aspergillus terreus* (No. GX7-3B)	*Bruguiera gymnoihiza* (Linn.)	South China Sea	MCF-7,	25.4 μM	Deng et al. 2013a

	3β,5α,14α-trihydroxy-(22*E*,24*R*)-ergosta-7, 22-dien-6-one **(112)**,	Known	Terrestial No cytotoxic , Marine cytotoxic	*Aspergillus terreus* (No. GX7-3B)	*Bruguiera gymnoihiza* (Linn.)	South China Sea	A549,	27.1 μM	Deng et al. 2013a
	3β,5α,14α-trihydroxy-(22*E*,24*R*)-ergosta-7, 22-dien-6-one **(112)**,	Known	Terrestial No cytotoxic , Marine cytotoxic	*Aspergillus terreus* (No. GX7-3B)	*Bruguiera gymnoihiza* (Linn.)	South China Sea	Hela and	24.4 μM	Deng et al. 2013a
	3β,5α,14α-trihydroxy-(22*E*,24*R*)-ergosta-7, 22-dien-6-one **(112)**,	Known	Terrestial No cytotoxic , Marine cytotoxic	*Aspergillus terreus* (No. GX7-3B)	*Bruguiera gymnoihiza* (Linn.)	South China Sea	KB	19.4 μM	Deng et al. 2013a
38	Nigerasterols A **(113)**	New		*Aspergillus niger* MA-132	*Avicennia marina*	Hainan, China	HL60	0.30 , 1.50 μM	Liu et al. 2013
	Nigerasterol B **(114)**	New		*Aspergillus niger* MA-132	*Avicennia marina*	Hainan, China	A549	1.82 , 5.41 μM	Liu et al. 2013
39	4-(methoxymethyl)-7-methoxy-6-methyl-1(3H)-isobenzofuranone **(115)**	New		*Penicillium* sp. ZH58	Mangrove endophytic fungus	South China Sea	KB and KB_{V200}	6 , 10 μg/mL	Yang et al. 2013a
40	*O*-glycoside, 3-*O*-(6-*O*-_-L-arabinopyranosyl)-_-D-glucopyranosyl-1,4-dimethoxyxanthone **(116)**	New		*Phomopsis* sp. (ZH76)	Excoecaria agallocha	Dong Sai of the South China Sea coast	HEp-2 and HepG2	9 and 16 μM	Huang et al. 2013
41	Botryosphaerin F **(117)**	New		*Aspergillus terreus* (No. GX7-3B)	*Bruguiera gymnoihiza* (Linn.)	South China Sea	MCF-7 and HL-60	4.49 and 3.43 μM	Deng et al. 2013b
	LLZ1271βCompound **(118)**	Known	Endophytes No Cytotoxic / , Marine cytotoxic	*Aspergillus terreus* (No. GX7-3B)	*Bruguiera gymnoihiza* (Linn.)	South China Sea	MCF-7 and HL-60	4.49 and 3.43 μM	Deng et al. 2013b

42	5-methyl-8-(3-methylbut-2-enyl) furanocoumarin **(119)**	New		*Penicillium* sp. ZH16	Mangrove tree Avicennia sp.	South China Sea	KB and KBv200	5 and 10 µg/ mL	Huang et al. 2012
43	Pullularin A **(120),**	known	From endopytes/ week cytotoxic	*Bionectria ochroleuca*	*Sonneratia caseolaris*	China	L5178Y	0.1 to 6.7 µg/mL	Ebrahim et al. 2012
	Pullularin C **(121),**	known	From endophytes/Cytotoxic / Marine cytotoxic	*Bionectria ochroleuca*	*Sonneratia caseolaris*	China	L5178Y	0.1 to 6.7 µg/mL	Ebrahim et al. 2012
	Verticillin D **(122),**	known	Mycoparasite Gliocladium catenulatum / Marine cytotoxic	*Bionectria ochroleuca*	*Sonneratia caseolaris*	China	L5178Y	0.1 to 6.7 µg/mL	Ebrahim et al. 2012
	Pullularins E and F **(123, 124)**	New		*Bionectria ochroleuca*	*Sonneratia caseolaris*	China	L5178Y	0.1 to 6.7 µg/mL	Ebrahim et al. 2012
44	Meroterpenes **(125-126)**	New		*Penicillium* sp. 303	Sea water	China	MDA-MB-435, HepG2, HCT-116, A549	Moderate cytotoxic activity (in the range of 11-37 µg/mL)	Li et al. 2014a
	Bisdiketone derivative **(127)**	New		*Penicillium* sp. 303	Sea water	China	MDA-MB-435	7.13 µM	Li et al. 2014a
45	Ditryptophenaline **(128)**	known	Terrestrial/ Marine cytotoxic	Endophytic fungus No·Gx-3a	Mangrove *endophytic fungus*	South China sea	KB, KBv200	8.0 µM and 12.0µM	Yang et al. 2013b
46	Compound **(129)**	New		*Phomopsis* sp. (No. SK7RN3G1	Mangrove sediment	Hainan, China	HEp-2 and HepG2	8 and 9 µg/mL	Yang et al. 2013c
47	2,3-didehydro-19α-hydroxy-14-epicochlioquinone B **(130)**	New		*Nigrospora* sp. MA75	*Pongamia pinnata*	Guangxi Zhuang Autonomous Region of China	MCF-7, SW1990, and SMMC7721	4, 5, and 7 µg/mL	Shang et al. 2012

48	1,4-dihydroxy-7-methylanthracene-9,10-dione **(131)**	known		Mangrove endophytic fungus No.5094	Unidentified Mangroove	South China Sea	KB, KBv200	LD_{50} values of 5.5,10.2 μM	Yang et al. 2013d
49	Scopararane I **(132)**	New		*Eutypella* sp. FS46	Marine sediment	South China Sea	MCF-7, NCI-H460 and SF-268	83.9, 13.5, 25.3 μg/ mL	Liu et al. 2016
	Pseurotin A **(133)**, Pseurotin D **(134)**	known	Terestrial endophytic cytotoxic / Marine cytotoxic	*Aspergillus* sp. (BRF 030)	Marine sediment	Northeast coast of Brazil	HCT-116	72.0, 85.0, 15.1 and 4.5 μM	Saraiva et al. 2015
50	Fumitremorgin C **(135)** and 12,13-dihydroxy Fumitremorgin C **(136)**.	known	Trrestrial Cytotoxic,/ Marine cytotoxic	*Aspergillus* sp. (BRF 030)	Marine sediment	Northeast coast of Brazil	HCT-116	72.0, 85.0, 15.1 and 4.5 μM	Saraiva et al. 2015
51	Tryptoquivaline T **(137)**	New		*Neosartorya fischeri*	Marine mud	Hainan Province of China	HL-60	82.3 μM	Wu et al. 2015
	Tryptoquivaline U **(138)**	New		*Neosartorya fischeri*	Marine mud	Hainan Province of China	HL-60	90.0 μM	Wu et al. 2015
	Fiscalin B **(139)**	Known	terestrial no cytotoxic	*Neosartorya fischeri*	Marine mud	Hainan Province of China	HL-60	8.8 μM	Wu et al. 2015
52	Penipacid A (**140)**	New		*Penicillium paneum* SD-44	Marine sediment	South China Sea	RKO	8.4 μM	Li et al. 2013
	Penipacid E **(141)**	New		*Penicillium paneum* SD-44	Marine sediment	South China Sea	RKO	9.7 μM	Li et al. 2013
	Compound **(142)**	known,	synthetic	*Penicillium paneum* SD-44	Marine sediment	South China Sea	HeLa	6.6 μM	Li et al. 2013
53	Prenpenicillide (**143)**	New		*Penicillium* sp. ZLN29	Marine sediment	Jiaozhou Bay of China	HepG2	9.9 μM	Gao et al. 2013

	Penicillide (**144**)	Know	Endophytic fungi Pestalotiopsis sydowiana , Proteosome inhibitor , Marine cytotoxic	*Penicillium* sp. ZLN29	Marine sediment	Jiaozhou Bay of China	HepG2	9.7 μM	Gao et al. 2013
54	Decumbenone C **(145)**	New		*Aspergillus sulphureus* KMM 4640	Marine sediment		SK-MEL-5	0.9 μM	Zhuravleva et al. 2012
55	6-O-methyl-7-chloroaveratin (**146**)	New		*Aspergillus* sp. SCSIO F063	Marine sediment	South China Sea	SF-268, MCF-7, and NCI-H460	7.1, 6.6, and 7.4 μM	Huang et al. 2012
56	Scopararane D (**147**)	New		*Eutypella scoparia* FS26	Marine sediment	South China Sea	MCF-7, NCI-H460, SF-268	IC_{50} 25.6-46.0 μM	Sun et al. 2012
	Libertellenone A (**148**) Diaporthein B (**149**)	Known	From *Eutypella scoparia* FS26	*Eutypella scoparia* FS26	Marine sediment	South China Sea	MCF-7, NCI-H460, SF-268	IC_{50} 4.4–20.0 μM	Sun et al. 2012
57	Cytochalasin K **(150),**	known	Terrestrial mycotoxin/ marine cytotoxic	*Arthrinium arundinis* ZSDS1-F3	*Phakellia fusca*	Xisha Islands of China	K562, A549, Huh-7, H1975, HL60, Hela, and MOLT-4	10.5 to 47.4 μM	Wang et al. 2015
	10-phenyl-[12]-cytochalasin Z16 **(151)**	known	Endophytes no cytotoxicity /	*Arthrinium arundinis* ZSDS1-F3	*Phakellia fusca*	Xisha Islands of China	K562, A549, Huh-7, H1975, HL60, Hela, and MOLT-4	1.13 to 18.8 μM	Wang et al. 2015
58	Aszonapyrone A **(152)**	known	Terrestrial / Marine cytotoxic	*Neosartorya fischeri* (KUFC 6344)	Coastal forest soil	Chonburi Province, Thailand	MCF-7 NCI-H460 and A375-C5	GI_{50} = 13.6, 11.6 and 10.2 μM.	Eamvijarn et al. 2013

	13-oxofumitremorgin B **(153)**	known	Terrestrial Cytotoxic	*Neosartorya fischeri* (KUFC 6344)	Coastal forest soil	Chonburi Province, Thailand	MCF-7 NCI-H460 and A375-C5	GI_{50} = 115.0, 123.31 and 68.6μM,	Eamvijarn et al. 2013
	Sartorypyrone A **(154)**	known	Marine/cytotoxic	*Neosartorya fischeri* (KUFC 6344)	Coastal forest soil	Chonburi Province, Thailand	A375-C5, MCF-7 NCI-H460	GI_{50}=21.5, 46.3 and 37.3 μM	Eamvijarn et al. 2013
	Sartorypyrone B **(155)**	known	Marine/cytotoxic	*Neosartorya fischeri* (KUFC 6344)	Coastal forest soil	Chonburi Province, Thailand	MCF-7 NCI-H460 and A375-C5	GI_{50}=17.8μM, 20.5μM and 25.0 μM	Eamvijarn et al. 2013
59	Disydonol A **(156)**	New		*Aspergillus* sp.	*Xestospongia testudinaria*	South China Sea	HepG-2 and Caski cancer	9.31 and 12.40 μg/mL	Sun et al 2012
	Disydonol C **(157)**	New		*Aspergillus* sp.	*Xestospongia testudinaria*	South China Sea	HepG-2 and Caski cancer	2.91 and 10.20 μg/mL	Sun et al 2012
60	Xestospongiamide **(158)**	New		*Xestospongia* sp.	Red Sea sponge Xestospongia sp	Jeddah, Saudi Arabia	Ehrlich ascites carcinoma and lymphocytic leukemia	5.0 μM	Ayyad et al. 2015
61	Marilines A1, A2 **(159, 160)**	New		*Stachylidium sp.*	Sponge *Callyspongia* cf. *C. flammea*	NA	HLE	0.86 μM	Almeida et al. 2012
62	4-(3-Hydroxyphenyl)-3-methoxyquinolin-2(1H)-one **(161)**	New		*Aspergillus versicolor* Y31-2	Seawater samples	Indian Ocean	MCF-7, SMMC-7721	16.6 and 18.2 μM	Li et al. 2016d
63	Penicilazaphilone B **(162)**	New		*Penicillium sclerotiorum* M-22	Rotted leaf sample	Hainan province, China	B-16	0.29, 0.44 and	Zhou et al. 2016
	Penicilazaphilone C **(163)**	New		*Penicillium sclerotiorum* M-22	Rotted leaf sample	Hainan province, China	SGC-7901	0.06, 0.72 μM	Zhou et al. 2016
64	5-hydroxymethyl-2-furancarboxaldehyde **(164)**	known Trrestrial		*Penicillium chrysogenum* HGQ6	Mud sample	Lianyungang sea	BGC823	0.19 μg/mL	Guo et al. 2016

65	Epiremisporine B **(165)**	New		*Penicillium purpurogenum* G59	Soil sample	Tideland of Bohai Bay around Lüjühe in Tanggu district of Tianjin, China,	K562, HL-60HL-60	69.0 and 62.9 µg/mL	Xia et al. 2015
	Epiremisporine B1 **(166)**	New		*Penicillium purpurogenum* G59	Soil sample	Tideland of Bohai Bay around Lüjühe in Tanggu district of Tianjin, China,	K562, HL-60HL-60	53.1 and 54.7 µg/mL	Xia et al. 2015
	Isoconiochaetone C **(167)**	New		*Penicillium purpurogenum* G59	Soil sample	Tideland of Bohai Bay around Lüjühe in Tanggu district of Tianjin, China,	K562, HL-60HL-60	IR% 20.4 and 26.0 at 100 µg/ mL	Xia et al. 2015
66	Penicitrinine A **(168)**	New		*Penicillium citrinum*	Marine-derived	Langqi Island, Fujian, China.	A-375, SPC-A1 and HGC-27	20.12 µM, 28.67 µM and 29.49 µM	Liu et al. 2015
67	Aspochalasin V **(169)**	New		*Aspergillus* sp	*Ligia oceanica*	Zhejiang Province of China	PC3 and HCT116	30.4 and 39.2 µM	Liu et al. 2014
68	Asperterrestide A **(170)**	New		*Aspergillus terreus* SCSGAF0162	*Echinogorgia aurantiaca*	Hainan Province, China	U937 and MOLT4	6.4 and 6.2 µM	He et al. 2013

69	Aculeatusquinone B **(171)**	New		*Aspergillus aculeatus*	Coral Reef	Similan Island, Phang Nga Province Thailand	HL-60, K562, A549	5.4-76.1 µM	Chen et al. 2013
	Aculeatusquinone D **(172)**	New		*Aspergillus aculeatus*	Coral Reef	Similan Island, Phang Nga Province Thailand	HL-60, K562, A549	5.4-76.1 µM	Chen et al. 2013
70	Diorcinol D **(173)**	New		*Aspergillus versicolor*	Mud sample	Yellow Sea	HeLa,K562	31.5 and 48.9 µM	Gao et al. 2013
	Diorcinol E **(174)**	New		*Aspergillus versicolor*	Mud sample	Yellow Sea	HeLa	36.5 µM	Gao et al. 2013
71	Chaunolidone A **(175)**	New		*Chaunopycnis* sp. (CMB-MF028)	*Siphonaria* sp.	Shorncliffe, Queensland	NCI-H460	0.09 µM	Shang et al. 2015
72	Penicimutalidine **(176)**	New		*Penicillium purpurogenum* G59	Soil sample	Tideland of Bohai Bay around Lüjühe in Tanggu district of Tianjin, China,	HL-60	95.2 µg/ mL, IR (%) value 20.8% at 100 mg/ml	Li et al 2016e
	Oxaphenalenone-SF226 **(177)**	known	Terrestrial parasitic fungus / Cytotoxic marine	*Penicillium purpurogenum* G59	Soil sample	Tideland of Bohai Bay around Lüjühe in Tanggu district of Tianjin, China,	K562	14.0 µg/ mL IR (%) 28.1% at 100 µg/ml	Li et al 2016e

73	Penicimutide **(178)**	New		*Penicillium purpurogenum* G59	Soil sample	Tideland of Bohai Bay around Lüjühe in Tanggu district of Tianjin, China,	HeLa	IR (%) 39.4% at 100 mg/ml	Wang et al. 2016
74	Oxalicumone A **(179)**	New		*Penicillium oxalicum* SCSGAF 0023	Gorgonian Muricella flexuosa	South China Sea	A375 and SW-620	11.7 and 22.6 μM	Sun et al. 2013
75	Indole alkaloid **(180)**	known	Synthetic no cytotoxic	*Aspergillus sydowii* SCSIO 00305	*Verrucella umbraculum*	Sanya, Hainan Province, China	A375	5.7 μM	He et al. 2012
76	AGI-B4 **(181)**	known	Terrestrial/ Cytotoxic marine	*Neosartorya fischeri* strain 1008F1	Marine-derived	Not Reported	SGC-7901, BEL-7404	0.29 μM, 0.31 μM	Tan et al. 2012
77	Chondrosterin J **(182)**	new		*Chondrostereum* sp	*Sarcophyton tortuosum*	Hainan Sanya National Coral Reef Reserve, China	CNE-1 and 2	1.32 and 0.56 μM	Li et al. 2014
78	(+)-6-*O*-demethylpestalotiopsin A and C **(183, 184)**	new		*Ascotricha* sp. ZJ-M-5	Mud sample	Zhejiang Province, China	HL-60 and K562	6.9 and 12.3 μM	Wang et al. 2014a
79	Pimarane diterpene (**185)**	new		Fungal strain HS-1	Sea cucumber, *Apostichopus japonicus*	Shandong Province, China	KB and KBv200	3.51, 2.34 μg/mL,	Xia et al. 2012
	Pimarane diterpene **(186)**	new		Fungal strain HS-1	Sea cucumber, *Apostichopus japonicus*	Shandong Province, China	KB and KBv200	20.74, 14.47 μg/mL,	Xia et al. 2012

	Diaporthins B **(187)**	known	Terrestrial mycotoxin / Marine cytotoxic	Fungal strain HS-1	Sea cucumber, *Apostichopus japonicus*	Shandong Province, China	KB and KBv200	and 3.86, 6.52 μg/mL	Xia et al. 2012
80	Trichodermamides B **(188)**.	known	Marine/cytotoxic	*Trichoderma virens*	Ascidian *Didemnum molle* and a green alga, *Halimeda* sp.	Papua New Guinea	HeLa	1.4-21 μM	Jans et al. 2017
81	Chromosulfine **(189)**,	New		*Penicillium purpurogenum* G59	Soil sample	Tideland of Bohai Bay around Lüjühe in Tanggu district of Tianjin, China,	K562, HL-60, BGC-823, HeLa and MCF-7	60.8, 16.7, 73.8, 75.4 and 59.2 μM	Yi et al. 2016
82	Neohydroxyaspergillic **(190)**	known	Terrestrial/ Cytotoxic marine	Fungus strain CF07002	Marine sediment	Eastern Pacific Ocean off Panama	Jurkat	60.2 μM	Cardoso-Martinez et al. 2015
	Neoaspergillic acid **(191)**	known	Terrestrial mycotoxin / Marine cytotoxic	Fungus strain CF07002	Marine sediment	Eastern Pacific Ocean off Panama	Jurkat, K562, U937, and Raji	31.6, 50.1, 42.6, and 54.9 μM	Cardoso-Martinez et al. 2015
83	Aspergiolide A **(192)**	New		*Aspergillus glaucus*	Marine sediment around the mangrove roots	Fujian province of the People's Republic of China.	BEL-7402	Targeting topoisomerase II	Wang et al. 2014b

84	Isosclerone **(193)**	Known	Terrestrial than marine	*Aspergillus fumigatus*	Marine green algae	Seosaeng-myeon, Ulsan in Republic of Korea	MCF-7	IC_{50} value 63 µM Also inhibits the protein and gene expressions of MMP-2,-9 in MCF-7 by altering MAPK signaling pathway	Li et al. 2014
851	Oxalicumone E **(194)**	New		*Penicillium oxalicum* SCSGAF 0023.	Gorgonian *Muricella flexuosa*	South China Sea	H1975, U937, K562, BGC823, MOLT-4, MCF-7, HL60 and Huh-7	< 10 µM	Bao et al. 2014
	Oxalicumone A (195)	known	Marine/cytotoxic	*Penicillium oxalicum* SCSGAF 0023.	Gorgonian *Muricella flexuosa*	South China Sea	H1975, U937, K562, BGC823, MOLT-4, MCF-7, HL60 and Huh-7	< 10 µM	Bao et al. 2014
86	Deoxybostrycin **(196)**	Known	Endophytes cytotoxic / Marine cytotoxic	*Nigrospora* sp. No. 1403	*Kandelia candel Mangrove endophytic fungus*	South China Sea	MDA-MB-435, HepG2 and HCT-116	3.19 , 29.99 and 5.69µM.	Chen et al. 2012
87	Auranomide A **(197)** Auranomide B **(198)** Auranomide C **(199)**	New		*Penicillium aurantiogriseum*	marine mud	Bohai Sea	K562 , ACHN, HEPG2 and A549	Moderate cytotoxic (Inhibition rate at 100 µg/mL) 5.8-20.48	Song et al. 2012
